# Immunomodulatory properties of probiotic extracellular vesicles- do they have the potential to fill the gap in therapeutic strategies for allergies?

**DOI:** 10.1186/s12964-025-02532-1

**Published:** 2025-11-26

**Authors:** Dominika Kozakiewicz, Agnieszka Razim, Irma Schabussova, Sabina Górska

**Affiliations:** 1 Laboratory of Microbiome Immunobiology, Hirszfeld Institute of Immunology and Experimental Therapy, Polish Academy of Sciences, Wroclaw, Poland; 2 Institute of Specific Prophylaxis and Tropical Medicine, Center for Pathophysiology, Infectiology and Immunology, Medical University of Vienna, Vienna, Austria

**Keywords:** Extracellular vesicle, Probiotic, Respiratory allergy, Immunomodulation, Tight junctions, TLR signaling

## Abstract

Probiotics, widely recognized for their health-promoting properties, have demonstrated efficacy in alleviating allergy symptoms. Recent research highlights that probiotic-derived extracellular vesicles (EVs) exhibit significant immunomodulatory potential, opening new avenues for therapeutic applications. While several immunoregulatory properties of probiotic EVs have been identified, their possible usage in anti-allergy strategies remains in its infancy. This review synthesizes current knowledge on probiotic EVs and explores their therapeutic potential as innovative immunomodulators for allergy prevention and treatment. Probiotic EVs facilitate host-microbiota communication by modulating immune signaling pathways, including receptor interactions, cytokine production, and regulation of tight junction proteins. Intriguingly, probiotic EVs show unique or sometimes even opposing properties compared to their parent bacteria, underscoring their potential as distinct therapeutic agents. Probiotic EVs offer a promising alternative to live bacterial therapies, particularly for vulnerable populations such as immunocompromised individuals and premature infants, where live probiotics may pose safety concerns. This review underscores the need for further research to elucidate the mechanisms of action, biological potential, and limitations of probiotic EVs, while addressing the challenges of translating these findings into effective anti-allergy strategies.

**Graphical abstract:**

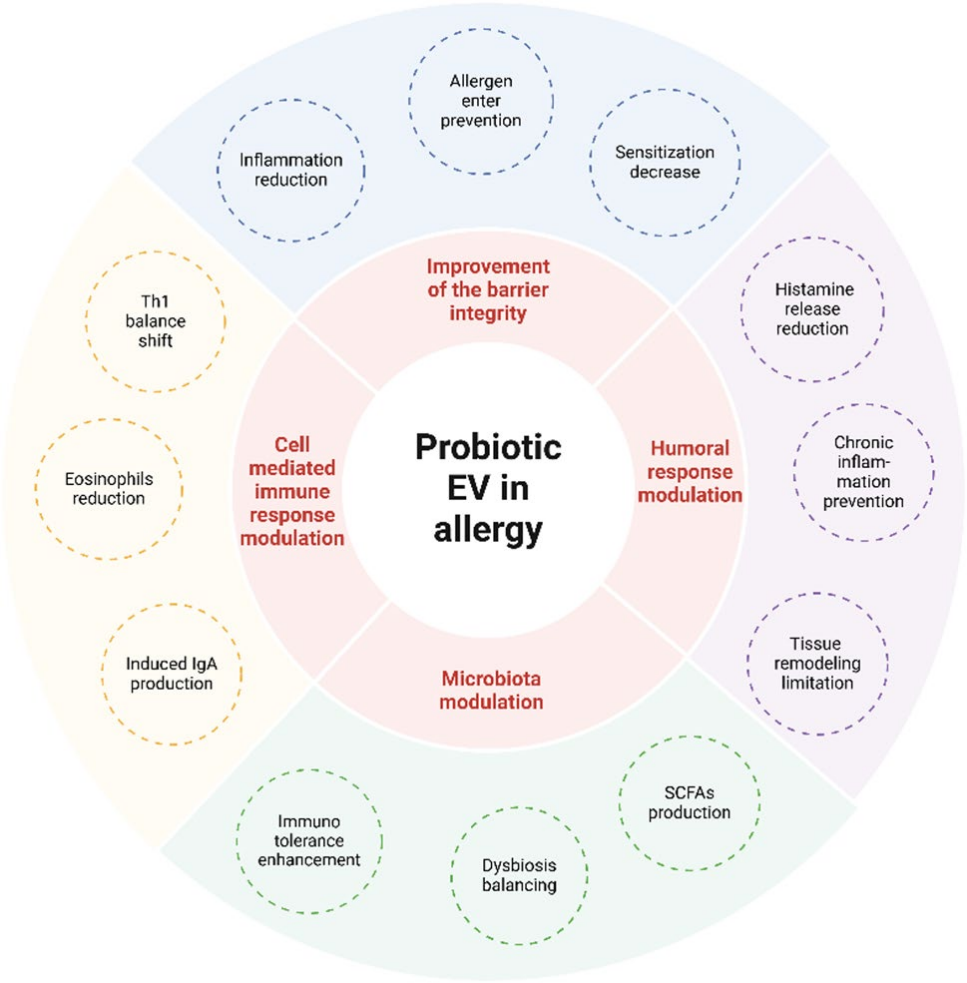

## Introduction

Allergy encompasses three prevalent allergic conditions which are allergic rhinitis (AR), atopic dermatitis (AD) food allergy (FA), and asthma. They are characterized by hypersensitive reactions to harmless substances like plant pollen or ingested food. AR is mediated by IgE antibodies [1] and is manifesting in the inflammation of nasal passages, while asthma in chronic inflammation and narrowing of the airways. FA affects more than 220 million people [2], whereas asthma affects approximately 334 million people worldwide [3]. Current allergy treatments predominantly rely on pharmacological interventions, including intranasal and oral antihistamines, intranasal, oral, and injectable corticosteroids, oral and intranasal decongestants, leukotriene receptor antagonists, oral cromolyn, intranasal anticholinergics and biologics, which often result in adverse effects such as fatigue and headaches. Another therapeutic possibility is immunotherapy, which involves repeated allergen injections, and biological therapies, particularly for conditions such as asthma [4]. However, these treatments can be ineffective for certain patients. Consequently, there is a critical need to develop a new generation of safe and effective allergy treatments.

Allergic sensitization begins with the first contact with the allergen, either through active epithelial damage caused by the allergen, or through a previously damaged barrier. Epithelial cells are equipped with several antigen receptors that, when activated, trigger a cascade of immune cells, leading to inflammation. Allergens that successfully cross the epithelial barrier are captured by antigen-presenting cells (APCs), which engulf and present the processed allergen to other immune cells through surface receptors. Under sensitization conditions, these processes result in the induction of Th2-prone lymphocyte T cells. Alternatively, Th2 cells can be activated by interleukin 4 (IL-4) secreted by group 2 innate lymphoid cells (ILC2s). Th2 cells produce IL-5, IL-9, and IL-13, which are responsible for the activation of eosinophils and B cells and the maturation of basophils [5]. Th2 cells stimulate B cells to produce allergen-specific IgE antibodies that bind to the Fc receptors on mast cells, which leads to activation of the cell and ultimately to its degranulation that is responsible for the typical symptoms of allergic reaction. The degranulation process is mediated by several kinases that activate the mitogen-activated protein kinase (MAPK) signaling pathway and phospholipase C, resulting in structural changes in the cytoskeleton [5]. The MAPK kinase pathway influences the expression of transcription factors and increases the level of produced prostaglandins. Along with the granules, several proinflammatory molecules, including histamine, proteoglycans, and serine proteases, which act locally to promote inflammation, are released from the cells. Moreover, activated mast cells produce several cytokines, including IL-4, IL-1, IL-3, IL-10, IL-13, tumor necrosis factor alpha (TNF-α), and TNF-β, which further fuel the inflammatory response. The process of hypersensitization has been described in detail by Jakubczyk et al. [5].

**Fig. 1:**
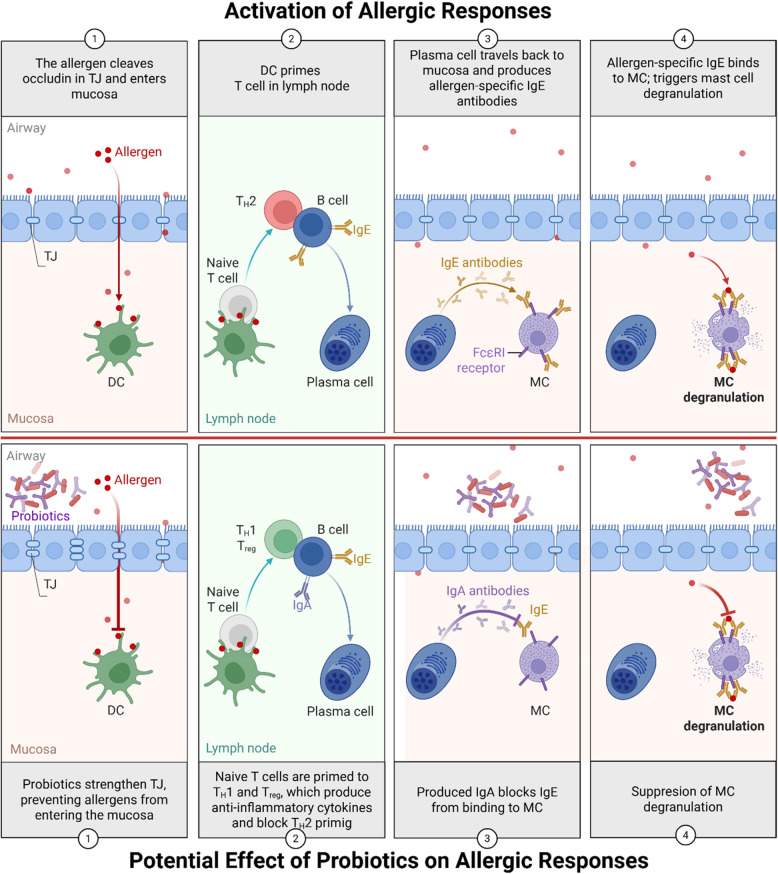
Schematic activation of allergic response and its modulation by probiotics. TJ, tight junctions; DC, dendritic cells; MC, mast cells; Th, T helper cells; Treg, T regulatory cells

The gut microbiota profoundly influences immune regulation and allergic sensitization, as studies highlight the strong interplay between the gut and lungs and reinforce the significance of the gut-lung axis [6, 7]. Consequently, strategies aimed at modulating microbial composition and activity- such as the use of probiotics and postbiotics- have gained significant attention as potential tools for allergy prevention and therapy (Fig. 1). The beneficial effects of probiotic supplementation in allergic diseases appear to be well supported in animal models [8–10]. However, it has to be underlined that these studies also have important limitations. Animal models of allergic responses induced with agents such as ovalbumin (OVA), recombinant birch pollens alergens (rBet), or house dust mite (HDM) do not capture the multifactorial nature of human allergy. Animal experiments represent simplified systems that exclude factors such as age, sex, hormones, microbiota, pollution, diet, epithelial damage, comorbidities, and medication use [11]. As a result, clinical trials on oral probiotics in allergic patients often yield less promising results than preclinical studies. Several clinical trials and meta-analyses suggest that selected probiotic strains may alleviate symptoms of seasonal or perennial allergic rhinitis, although the magnitude of benefit is usually modest and depends strongly on the strain, dosage timing of administration (prenatal, infancy, or adulthood), and the allergy phenotype of the studied population [12–15].

Emerging evidence implicates the gut–skin axis in AD: disturbances in the gut microbiota (dysbiosis) have been associated with the onset, severity, or persistence of AD [16, 17]. For instance, infants who later develop eczema/AD often show reduced gut microbial diversity or altered recruitment of specific taxa (e.g. lower *Bifidobacterium*, *Lactobacillus*, or increased *Clostridioides*) early in life. In one study, the composition of the gut microbiome in the first week of life correlated with eczema incidence in the first year [18]. Systematic reviews and meta-analyses in adults also report significant differences in gut microbiota profiles in AD patients compared to healthy controls, though results are heterogeneous [19, 20].

The most commonly used probiotics belong to the genera *Lactobacillus (L.)*,* Lacticaseibacillus (L.)*,* Lactiplantibacillus (L.)*, *Limosilactobacillus (L.)*, and *Bifidobacterium (B.)*. Although probiotics are generally considered as safe, people with a weakened immune system, such as premature babies, chronically ill individuals, or the elderly, may be at risk of infection, antibiotic resistance transfer, or even bacteremia [21–24]. Therefore, alternative therapies based on nonliving bacteria and their compounds are needed [25].

Probiotic bacteria produce various bioactive molecules- collectively known as postbiotics- such as bacteriocins, short-chain fatty acids (SCFAs), exopolysaccharides, and cell-wall components, many of which exert immunomodulatory effects. Bacteriocins (e.g., nisin, plantaricin, sublancin) interact with epithelial and immune cells, modulating Toll-like receptors (TLR), NF-κB, and MAPK pathways to promote anti-inflammatory cytokine profiles. SCFAs (acetate, propionate, butyrate) act through G-protein–coupled receptors and histone deacetylase inhibition, enhancing Treg differentiation, IgA production, and M2 macrophage polarization, thus supporting mucosal tolerance and barrier integrity [26]. For example, the allergy-alleviating properties of heat-inactivated *L. casei* Shirota [27], heat-inactivated *B. longum* ssp. *longum* CCM 7952 [28], the polysaccharide BAP.1 from *B. adolescentis* CCDM 368 [29], the polysaccharide B. PAT from *B. animalis* ssp. animalis CCDM 218 [30] or exopolysaccharides isolated from *L*. *rhamnosus* LOCK900 [31] have already been demonstrated. A relatively new line of research is the use of extracellular vesicles (EVs) produced by bacteria [32].

In 1966, electron microscopy revealed the existence of outer membrane vesicles (OMVs) in gram-negative *Escherichia coli* (*E. coli*), marking the first observation of EVs produced by bacteria [33]. The primary components of gram-negative bacterial EVs include lipopolysaccharide (LPS), periplasmic proteins, lipids, and nucleic acids (DNA and RNA), some of which function as virulence factors [34, 35]. EV cargo can be luminal and surface-attached [36], and specific to the species that it comes from [37]. The first gram-positive bacterial EVs were identified almost 30 years later [33, 38]. Like Gram-negative EVs, they carry a wide range of cargo molecules, including nucleic acids, proteins, and lipids, but they uniquely feature lipoteichoic acid (LTA) and cytoplasmic proteins [39]. The production mechanism of Gram-positive EVs involves cell wall-degrading enzymes that weaken the bacterial cell wall, which makes EV release possible [40]. The composition of bacterial EV cargo is already well described: comprehensive proteomic, lipidomic and nucleic-acid profiling studies have mapped recurring cargo classes (LPS/LTA, outer-membrane and cytosolic proteins, enzymes, toxins, metabolites, DNA and diverse RNA species) across multiple Gram-negative and Gram-positive species, and functional work has demonstrated roles for these cargo molecules in virulence, immune modulation, interbacterial communication and host signaling [41–44]. Large-scale reviews and recent surveys synthesize these molecular catalogues and the experimental methods used (proteomics, lipidomics, sequencing and biochemical assays), making the EV cargo repertoire one of the best-characterised aspects of bacterial extracellular vesicle biology [45, 46].

Products secreted by probiotic bacteria, including EVs, have significant biological effects on the host organism, as demonstrated in numerous in vivo and in vitro studies [47–49] summarized in Table 1. The scientific interest in EVs stems from the several advantages that EVs offer over live bacteria. Their smaller size facilitates dissemination and migration to distant tissues and interaction with immune cells, which might be difficult for whole bacteria [50, 51]. As mentioned above, the use of EVs avoids the safety concerns associated with live bacterial strains [52]. To date, EVs have been successfully isolated from probiotic strains such as *E. coli* Nissle 1917 [49], *E. coli* ECOR12, ECOR63, ECOR53, and ECOR12 [53], *E. coli* O83 [32], *Akkermansia muciniphila* ATCC BA-835 [54], *Bacteroides vulgatus* mpk [55], *Bacteroides fragilis* NCTC9343 [56], *B. acidifaciens* BNCC353574 [57], *B. bifidum* LMG13195 [58], BIA-7 [59], *B. longum* KACC 91,563 [50], *C. butyricum* [60], *L. kefir* KCTC 3611, *L. kefiranofaciens* KCTC 5075, *L. kefirgranum* KCTC 5086 [61], *L. murinus* [62], *L. rhamnosus* JB-1 [63], *L. plantarum* KCCM11179P [64] and Q7 CP019712-16 [65], *L. plantarum* subsp. *plantarum* NBRC 15,891 [66], *L. paracasei* [67], *L. sakei* NBRC15983 [68], *L. reuteri* BBC3 [69], *L. ameliorate* PRCC-1301 [70], *L. casei* BL23 [40], CRL431 [71], *Faecalibacterium prausnitzii* A2-165 [72], and *Propionibacterium freudenreichii* CIRM-BIA129 [73, 74]. Many of these bacteria are already well characterized in terms of allergy treatment and prevention.

**Table 1:** Biological effects of EVs

Bacteria	EV-host interaction			Ref.
	Induced cytokines	Induced gene/protein expression	Other activity	
*E. coli* O83	IL-6, IL-8, INF-γ, IL-12, IL-18, TNF-α, IL-10, IL-12p40, IL-4, IL-1β↑		- strengthening the TJs; - modulation of the innate immune system by NOD1-mediated activation of NF-κB; - maturation of dendritic cells; - activation of macrophages and induction of phagocytosis via NO and iNOS; - promoting immunity maturation;	(32,49,53,88,111,112,118,121)
*E. coli Nissle*				(49)
*E. coli Nissle*,* E. coli ECOR63*				(53)
*E. coli Nissle*,* E. coli ECOR12*				(88)
*E. coli Nissle*,* E. coli ECOR12*,* ECOR63*,* ECOR53*				(110)
*E. coli Nissle*,				(111)
*E. coli Nissle*,* E. coli ECOR12*				(121)
*A. muciniphila* ATCC BA-835	IFN-γ ↑ IL-6, TNF-α ↓	*ppar-α*,* ppar-γ*↑ *tlr4*,* tlr2*,* tlr5*,* tlr9*↓	- strengthening the TJ, increasing the number of anti-inflammatory M2 macrophages;	(54,90)
*B. vulgatus* mpk	TNF-α, IL-6↓		- semimaturation of DCs;	(55)
*B. fragilis* NCTC9343	IL-10↑	FoxP3↑	- induction of Treg cells;	(56)
*B. acidifaciens* BNCC353574			- strengthening the TJs;	(57)
*B. bifidum* LMG13195	IL-10↑	FoxP3↑	- induction of Treg cells;	(58)
*B. bifidum* (BIA-7)			- strengthening the TJs;	(59)
*B. longum* KACC 91,563			- induction of mast cell apoptosis;	(50)
*C. butyricum*			- affecting gut microbiotoa;	(60)
*L. kefir* KCTC 3611, *L. kefiranofaciens* KCTC 5075, and *L. kefirgranum* KCTC 5086	IL-8, TNF-α↓		- blocking the phosphorylation of p65 (subunit of NF-κB);	(61)
*L. murinus*			- strengthening the TJs;	(62)
*L. rhamnosus* JB-1			- TLR2 activation;	(63)
*L. plantarum* KCCM11179P, Q7 CP019712-16	IL-10, GM-CSF↑ IL-6, IL-4, TNF-α, IL-2, IL-1β↓		- increasing the number of anti-inflammatory M2 macrophages;	(64,65)
*L. paracasei*	IL-10, TGF-β↑ TNF-α, IL-1α, IL-1β, IL-2, IL-8↓		- inhibiting JNK phosphorylation; - reducing airway resistance;	(67,119)
*L. sakei* NBRC15983	IL-6↑	IgA↑		(68)
*L.s reuteri* BBC_3_	IL-10, TGF-β↑ IFN-γ, IL-17↓		- inhibiting NF-κB activation;	(69)
*L. ameliorate* PRCC-1301	IL-2, IL-8, TNF-α↓		- inhibiting NF-κB activation; - strengthening the TJs;	(70)
*L. casei* BL23, CRL431	IL-4, IL-10↑	*tlr9*↓	- activating the phosphorylation of EGFR; - reducing the levels of phosphorylated Akt and ERK1/2;	(71,100)
*F. prausnitzii* A2-165	IL-10, IL-4, IL-8, TGF-β2, TNF-α ↑ IL-6, IL-1, IL-2, IL-12, IL-17, TNF-β,α↓	*tlr7*,* tlr3*, *IL-1Ra*, ↑	- strengthening the TJs; - lymphotoxin alpha↓	(72,92,101)
*L. lactis*	INF-γ, IL-12↑ IL-5, IL-13↓	GATA-3↓	- lowering eosinophils count in BAL fluid; - increasing Th1 type response; - reducing STAT6 phosphorylation	(120)
*P. freudenreichii* CIRM-BIA129	IL-8↓		- reducing LPS-dependent NF-κB activation.	(73)

Given the limited number of studies on the direct use of EVs in allergy treatment, this review systematically evaluates the current understanding of the immunomodulatory features of probiotic EVs. Furthermore, we critically examine the modulatory properties relevant to allergy therapy and delineate the mechanisms by which EVs may restore immune balance and strengthen the mucosal barrier.

##  Interaction of probiotic-derived EVs with epithelial cells

### Probiotic-derived EVs are recognized by receptors on epithelial cells

In addition to serving as the first line of defense, epithelial cells have a regulatory function, sensing the environment and activating various immunological pathways via a wide range of pattern recognition receptors (PRRs). These receptors include TLR 1–10, nucleotide-binding leucine-rich repeat-containing receptors (NLRs), retinoic acid-inducible gene I (RIG-I)-like receptors (RLRs), dectins, damage-associated molecular pattern receptors (DAMPs), and protease-activated receptors (PAR) [75]. When allergens are recognized by these receptors, epithelial cells release chemokines, cytokines, and danger signals that activate and recruit immune cells, e.g., dendritic cells, macrophages, or ILC2s, to the site of action [75, 76]. The widely supported hygiene hypothesis of the increase in allergy incidence over past decades continues to be supported by studies suggesting a protective role of PRR activation, especially TLR activation. These receptors play a role in the pathogenesis of allergic diseases by influencing the activity of T regulatory (Treg) cells; the differentiation of Th17, Th1, and Th2 lymphocyte subpopulations; cytokine production by mast cells; and the activation of eosinophils [77].

TLR stimulation is a key factor in the development and modulation of allergic responses [28, 78–80]. In individuals with allergies, the nasal epithelium shows significantly increased expression of TLR2 (the receptor for lipoteichoic acid, LTA) and TLR4 (the receptor for lipopolysaccharides, LPS), suggesting that these receptors play a crucial role in recognizing allergens and initiating immune response [81]. Similarly, the expression of TLR3 (which detects double-stranded RNA (dsRNA)) is upregulated in allergic individuals, with its expression increasing in lymphoid tissues upon exposure to allergens such as OVA. These findings indicate that TLR3 is integral in modulating asthma and other allergic conditions [82, 83]. Moreover, in patients with AR, there is an increased expression of TLR7 (single-stranded RNA receptor (ssRNA)) and TLR9 (DNA receptor) in basophils, further underscoring the importance of TLR-mediated signaling in allergy development [84, 85]. However, there is little information on whether changes in TLR expression in allergic individuals are a cause or effect of allergy. Worth noting is that the beneficial effects of some probiotics, particularly through their production of short-chain fatty acids (SCFAs), may be linked to their ability to modulate TLR signaling. SCFAs have been shown to decrease the activity of NF-κB, a key transcription factor in inflammation, and inhibit the release of proinflammatory cytokines such as IL-8 and TNF-α [86]. Through the modulation of TLR signaling pathways, these probiotics could reduce the severity or incidence of allergic reactions [87].

Probiotic EVs have been shown to activate TLRs and elucidate various modulatory effects. BALB/c mice fed with *L. rhamnosus* JB-1-derived EVs presented greater TLR2 activation than control mice did, although to a smaller degree than that observed following the consumption of *L. rhamnosus* JB-1 bacteria [63]. The probiotic *E. coli*-derived EVs, which contain peptidoglycan (PG), activated NOD1 and NOD2 receptors in Caco-2 cells [88]. The activation of NOD receptors might be crucial for the production of certain cytokines, as has been shown for *E*. *coli* ECOR12-derived EVs [88]. They upregulated *il-6* and *il-8* mRNA expression in Caco-2 cells, which was confirmed to be NOD1-dependent by using NOD1-knockout cells. The authors also confirmed that EVs caused the activation of NF-κB, hence inducing the transcription of target proinflammatory genes [88, 89]. Recently, Schmidt et al. reported that both *E. coli* O83-derived vesicles and parent bacteria activate TLR4 and TLR2 receptors in HEK-293 TLR reporter cells. However, only whole bacteria are able to activate the TLR5 receptor, which recognizes flagellar proteins [32]. Interesting observations have been made in studies of *(A) muciniphila* (ATCC BAA-835) and its AmEVs [54], suggesting that interactions with cell surface receptors might sometimes be completely different for the parent bacteria and their EVs. In the LPS-activated Caco-2 cell line, whole bacteria increased the expression of *tlr2* and *tlr4* due to the presence of the Amuc-1100 protein or LPS, respectively. However, AmEVs had the opposite effect, as they decreased the expression of the *tlr2* and *tlr4* genes [54]. Surprisingly, in a human hepatic stellate cell line, AmEVs significantly reduced the expression of other receptor genes, namely, *tlr5* and *tlr9* [90]. The TLR2 receptors on RAW264.7 cells were shown to recognize *(B) longum* EVs, which resulted in the stimulation of IL-6 production via the activation of NF-κB [91]. Furthermore, the same effect was induced by the addition of the recombinant lipoprotein ESBP, a substrate-binding protein associated with the nickel/oligopeptide ABC import system, which is present on EVs [91]. Strikingly, bacterial EVs can also activate intracellular TLR3 or TLR7. Both intact *F. prausnitzii* and its EVs upregulated *tlr3* expression, but only EVs increased the expression of *tlr7.* There was no change in the expression of the *tlr1*,* tlr2*,* or tlr4* genes in Caco-2 cells [92]. The expression of *tlr7* may be activated by the presence of RNA in the EV cargo since bacterial EVs are packed with small noncoding RNA [93]. For example, EVs obtained from *L. plantarum* JCM 8341 contain RNAs, with the majority being small RNAs such as 5 S RNA, as well as tRNAs and some 16 S and 23 S rRNAs. EV-derived small RNAs seem to have important biological activity, since small RNAs isolated from *L. plantarum* NBRC 15,891-derived EVs inhibited LPS-induced inflammation in HT29 cells in a concentration-dependent manner [66].

Probiotic-derived EVs may modulate allergic disease by engaging PRRs and signaling pathways like NF-κB and MAPK, reprogramming mucosal immunity to favor tolerance and barrier function. By activating TLRs and NOD receptors, EVs enhance barrier defenses and mucin production, while EV cargoes such as small RNAs shift cytokine profiles toward regulatory or Th1 responses, reducing Th2 polarization. Their nanoscale size and lack of viability allow EVs to reach mucosal and systemic sites efficiently, highlighting their promise as immunomodulatory agents.

### Probiotic-derived EVs affect the epithelial barrier by influencing the expression of tight junction proteins

The epithelium plays a key role in maintaining of the proper function of the intestinal and respiratory tracts, forming the first physiological barrier against the invasion of pathogens and the infiltration of allergens. Most of the foreign particles are trapped by a thick mucus layer, e.g., in the intestine, or are removed by ciliary movements of the respiratory tract. TJ proteins, which are cell- cell junctional complexes located on the apical side of epithelial cells, are vital for maintaining the integrity of the epithelial barrier. Impaired TJ structures are observed in patients with asthma, atopic dermatitis, and allergic rhinitis, suggesting that epithelial barrier dysfunction may contribute to the initiation or progression of allergic diseases [94]. Allergens and pollutants can damage TJs [95, 96], increasing paracellular transport in epithelial cells and allowing allergen delivery into subepithelia [65, 67]. Damaged epithelial cells produce alarmins IL-25, IL-33, and thymic stromal lymphopoietin (TSLP), which alert and activate immune cells to neutralize foreign molecules [45], but its excessive activation can lead to hypersensitivity. Numerous experimental studies have shown the positive effect of probiotic bacteria on epithelial barrier enhancement [86]. Selected probiotics increase the level of gene expression and redistribution of TJ proteins such as zonula occludens-1 (ZO-1), occludin (OCLN), and claudins (CLDNs) in epithelial cells [86]. Probiotics exert their beneficial effects through surface antigens or small metabolites like short fatty acids [97], which can cross epithelial barriers and have local or distant effects. Recently, it has been shown that, similarly to bacteria, EVs also regulate epithelial integrity. EVs produced by *L. kefirgranum* PRCC-1301 reduced epithelial barrier permeability both in vitro in DSS-treated Caco-2 cells and in vivo in a mouse model of DSS-induced colitis by recovering the loss of ZO-1, CLDN-1, and OCLN in colonic epithelial cells [70]. EVs derived from a different strain of lactobacilli, *L. murinus*, had a protective effect on mice challenged with deoxynivalenol (DON), a common environmental pollutant [62]. The TJ barrier (ZO-1 and OCLN) was strengthened in DON-treated epithelial cells cocultured with probiotic EVs-pretreated macrophages [62]. A similar effect was observed using *B. acidifaciens* BNCC353574 and its EVs in a mouse model of DSS-induced colitis [57]. The simultaneous administration of bacteria or EVs with DSS decreased the degradation of ZO-1 and OCLN [57]. Other EVs, such as those produced by *A*. *muciniphila* ATCC BA-835 (AmEVs), reduce epithelial barrier damage in LPS-treated Caco-2 cell cultures [54]. The mechanism is thought to be based on the ability of AmEVs to increase OCLN expression through the activation of AMP-activated protein kinase (AMPK) [54]. Other studies have shown that AmEVs have a positive dose-dependent effect on mRNA expression and upregulate the expression of TJ proteins in the human hepatic stellate (LX-2) cell line [90]. EVs isolated from the probiotic *E. coli* Nissle 1917 (*Ec*N*)* upregulate ZO-1 and CLDN-14 and downregulate CLDN-2 protein expression in the Caco-2 cell line, which was confirmed both by RT-qPCR and Western blot [53]. Moreover, the modulatory effect of EVs is not always the same as that of whole bacteria, as the induction of CLDN-14 was attributed to the Tcpc protein secreted by the bacterium, whereas for OMVs, this effect was shown to be Tcpc-independent. It was also reported that *Ec*N EVs, in contrast to the cell lysates, induce IL-22 expression in human colonic explants, that is responsible for reinforcing the intestinal barrier, thus limiting the access of microbial compounds and allergens to the systemic circulation [98]. Hassani et al.. reported that both *B*. *bifidum* BIA-7 whole bacterium and its EVs affect epithelial TJs by increasing the expression levels of selected transcription factors in Caco-2 cells [59]. *B. bifidum* BIA-7 significantly upregulated *ahr* and *hes-1* expression and slightly increased *notch-1* gene expression. In contrast, EVs increase the expression of *cyp1a1*,* zo-1*, and *ocldn*, improving epithelial permeability. EVs of *Faecalibacterium prausnitzii* A2-165 are another example confirming that postbiotics might have different immunomodulatory properties than whole parent bacteria. These EVs, but not whole bacteria, increased the expression of the *zo-1* and *ocldn* genes in a dose-dependent manner in the Caco-2 cell line [72].

In conclusion, probiotic-derived EVs enhance epithelial barrier integrity by upregulating tight junction proteins and mucosal defenses, limiting allergen entry and reducing Th2-driven allergic responses. By supporting epithelial repair and decreasing antigen burden, EVs promote tolerogenic immune behavior and Treg induction, shifting mucosal immunity toward tolerance. Their effects are strain- and cargo-specific and vary with composition, dose, and host context.

###  Probiotic-derived EVs modulate cytokine production in epithelial cells

EVs from different bacterial strains interact with epithelial cells in multiple ways, and their combination may have an enhanced immunomodulatory effect. The anti-inflammatory effects of kefir grain *Lentilactobacillus-*derived EVs (*L. kefir* KCTC 3611, *L. kefiranofaciens* KCTC 5075, and *L. kefirgranum* KCTC 5086) were studied in TNF-α-treated Caco-2 cells [61]. Compared with single-strain EVs, mixtures of EVs from all three strains were more effective in reducing IL-8 levels, which was associated with the inhibition of p65 phosphorylation (subunit of NF-κB). In an in vivo model of TNBS-induced IBD, EVs alleviated inflammation by reducing transmural leukocyte infiltration and lowering the serum levels of myeloperoxidase (MPO) [61]. Kefir grain *L. ameliorate* PRCC-1301-derived EVs decreased the expression levels of the proinflammatory cytokines IL-2, IL-8, and TNF-α in DSS-induced Caco-2 cells via the inhibition of NF-κB activation [70]. *L. paracasei*-derived EVs decreased the expression of the proinflammatory cytokines IL-1α, IL-1β, IL-2, and TNF-α and increased the expression of IL-10 and TGF-β in LPS-treated HT29 cells [67]. The anti-inflammatory effect was associated with the suppression of *cox-2* and *iNOS* expression and the inhibition of IκB phosphorylation, which regulates the expression of inflammation-associated proteins. Lactobacilli EVs were also found to affect endoplasmic reticulum (ER) stress, which has been implicated in many diseases, including obesity, diabetes, inflammatory diseases, and neurodegenerative disorders [67]. It is not yet known which EV components are responsible for specific immunomodulatory effects. Like parent bacteria, EVs produced by *L. reuteri* BBC_3_ attenuated intestinal inflammation in LPS-treated chickens by increasing the levels of IL-10 and TGF-β, while decreasing the expression of proinflammatory genes in the chicken jejunum [69]. This effect was shown to be dependent on the content of vesicular nucleic acids and proteins. Proteomic studies have shown that EVs containing proteins such as glucosyltransferase, serine protease, and elongation factor Tu mediate probiotic benefits [69]. The anti-inflammatory effect of EVs from *P. freudenreichii* CIRM-BIA 129 was shown to be partly dependent on the SlpB surface protein [73]. Compared with the wild-type strain, the ΔslpB mutant only partially reduced NF-κB activation in LPS-treated HT29 cells, which lowered its activity to a level comparable to that of the negative control. *L. casei* BL23-derived EVs contain P40 and P75 proteins bound to their surface, which can activate the phosphorylation of epidermal growth factor receptor (EGFR), which protects the intestinal epithelium from induced inflammation. Additionally, EVs reduce the protein levels of phosphorylated Akt and ERK1/2, which are responsible for inflammation signaling via multiple pathways [40, 99, 100]. Bacteria and their EVs may share some common immunomodulatory features. However, they may also differ significantly, as in the case of *F. prausnitzii* A2-165 (DSM 17677). Bacteria induce the production of factors such as FOS, JUN, TNF-α, and NF-κB1, whereas EVs upregulate CXCL8, CCL2, FOS, MAP2K4, IRF1, NF-κB1A, and TNF-α. Both significantly increased the production of IL-4, IL-8, and IL-10 and significantly decreased the production of IL-1, IL-2, IL-6, IL-12, IL-17, and IFN-γ. However, compared with parent bacteria, EVs are more potent at decreasing proinflammatory cytokines and increasing anti-inflammatory cytokines [92]. In other studies, the bacterial culture supernatant from *F. prausnitzii* A2–165 (DSM 17677) upregulated bone morphogenetic proteins 4, 6, 7 (BMP4,6, 7), complement component 5 (C5), chemokine (C-C motif) ligand 20 CCL20, ciliary neurotrophic factor (CNTF), colony stimulating factors 1, 3 (CSF1, CSF3), chemokine (C-X-C motif) ligands 2, 9 (CXCL2, CXCL9), IL-10, IL-11, IL-12 A, IL-15, IL-27, IL-3, leukemia inhibitory factor (LIF), lymphotoxin beta (LTB), macrophage migration inhibitory factor (MIF), myostatin, and tumor necrosis factor (ligand) superfamily, member 10 (TNFSF10) production in lung adenocarcinoma epithelial cells (A549), whereas isolated EVs significantly increased only the production of the anti-inflammatory cytokines IL-10 and TGF-β2 and decreased the production of the proinflammatory cytokines IL-6, TNF-β, and TNF-α [101].

These studies collectively indicate that probiotic-derived EVs carry multiple bioactive components that suppress key proinflammatory pathways and therefore have clear potential to prevent or attenuate allergic disease. By inhibiting NF-κB signaling and lowering proinflammatory cytokines (TNF-α, IL-1, IL-6, IL-8) while increasing anti-inflammatory mediators (IL-10, TGF-β), EVs reduce the local tissue inflammation that amplifies Th2 responses and allergic effector mechanisms. EV-mediated upregulation of barrier-protective programs (e.g., ZO-1, occludin, claudins, and IL-22) and activation of repair pathways (AMPK, EGFR) can limit allergen translocation across mucosae, decreasing antigen loading to dendritic cells and the likelihood of sensitization. The enrichment of tolerogenic signals (IL-10, TGF-β) and the evidence that EVs favor regulatory phenotypes (Tregs, M2 macrophages) imply a shift of adaptive immunity away from pathogenic Th2 polarization and IgE production. EV cargo such as P40/P75, SlpB, and small RNAs appear to mediate many of these effects, suggesting that defined vesicle components could be harnessed as targeted, non-viable immunomodulators. Because EVs are nanoscale and nonliving, they may reach mucosal and systemic immune sites more readily than whole bacteria, enabling localized or systemic delivery strategies (oral, nasal, or topical) for allergy prevention or adjunctive therapy.

## Regulation of immune response by probiotic-derived EVs via interaction with apcs

###  In vitro studies

The epithelial barrier function is supported by immune cells such as granulocytes, lymphocytes, macrophages, dendritic cells (DCs), and mast cells that are in the immediate vicinity of basal cells [102]. In the context of allergies, APCs such as macrophages and DCs play a decisive role in the development of this disease. Upon exposure to local microenvironments, including allergens, recruited macrophages can be polarized into either classically activated (M1) or alternatively activated (M2) phenotypes, which have a profound impact on asthma pathogenesis [103]. During allergy development, DCs can be activated by a variety of TLR receptors, such as TLR5, a receptor for flagellin (a bacterial protein shown to be present in house dust mite extracts [104]), or TLR4, which can be activated by a variety of airborne allergens [105, 106]. Other DCs receptors are mannose receptors that are type-I integral transmembrane proteins which activation results in the upregulation of indoleamine-pyrrole 2,3-dioxygenase (IDO, an enzyme that participates in tryptophan metabolism). The activity of IDO is crucial in maintaining the balance between Th1 and Th2 cells [107]. Disturbance of the delicate immune balance ultimately leads to the development of allergies, and intensive work is ongoing to reverse this process through specific interactions of bacterial EVs with immune system cells.

EVs’ specific cargo is responsible for the cellular response upon successful delivery to host cells [108]. To date, EVs have been shown to be engulfed by eukaryotic cells through clathrin-mediated endocytosis, the lipid raft-mediated pathway, actin-driven pinocytosis, and phagocytosis [109]. EVs, via their specific cargo, modulate immune cells, influencing their maturation, differentiation, and production of Th1-, Th2- and Treg-specific cytokines [110]. For example, EVs derived from *L. rhamnosus* JB-1 are engulfed by endocytosis into intestinal epithelial cells both in vitro and in vivo, likely through a clathrin-mediated process; however, because of the presence of lipoteichoic acid, they also interact with the DC surface receptor TLR2, which results in IL-10 expression [63].

*E. coli*-derived EVs were shown to induce the maturation of DCs. Compared with untreated cells, monocyte-derived DCs (mo-DCs) exposed to EVs isolated from probiotic, commensal, and pathogenic *E. coli* strains (10 µg/ml) expressed high levels of the maturation surface marker CD83 and a reduced level of CD209 [111] on their surface. Compared with EVs isolated from commensal, or pathogenic *E. coli* strains, probiotic *E. coli* Nissle-derived EVs induced higher levels of Th1-related cytokines (INF-γ, IL-12, and IL-18) in human mo-DCs. In the same study, Diaz-Garrido et al.. used an *E. coli* Nissle capsular polysaccharide mutant to show that produced EVs do not induce maturation or cytokine production; however, they did not prove the polysaccharide presence/absence in any of the tested EV preparations [111]. In their next work, Diaz-Garrido et al.. reported that DCs induced with probiotic *E. coli* EVs release immune mediators that are able to communicate with T cells without a direct contact [110].

Studies have shown that probiotic EVs modulate the activity of macrophages in an in vitro model of LPS-induced inflammation. Compared with inactivated bacteria, RAW 264.7 macrophages pretreated with *L. paracasei*-derived EVs and treated with LPS had significantly lower TNF-α secretion and NO production [67]. Similarly, Hu et al. showed that preincubation with *L*. *reuteri* BBC3-derived EVs inhibited NF-κB activity and downregulated the expression of TNF-α, IL-1β, and IL-6 in HD11 cells treated with LPS [69]. Without LPS treatment, these EVs upregulated the expression of IL-10 and TGF-β in macrophage culture. Hu et al. also showed that in a coculture of EV-pretreated macrophages and splenic lymphocytes, the latter also expressed high levels of the IL-10 and TGF-β genes, whereas the expression of the IFN-γ and IL-17 genes was downregulated compared with that in the control [69]. These findings support the view that EVs can suppress the proinflammatory response of macrophages and, indirectly, lymphocytes derived from the spleen [69]. Moreover, they have shown that this effect depends on the protein and nucleic cargo of the EVs. Furthermore, probiotic *E. coli* Nissle-derived EVs were shown to activate macrophages and phagocytosis. EVs internalization is followed by NO production, which is mediated by inducible nitric oxide synthase (iNOS), and by enhanced macrophage phagocytic function through improved acid phosphatase (ACP) activity. Additionally, EVs at the concentration of 1.0 µg/ml also significantly induced the production of pro-inflammatory IL-6, TNF-α, anti-inflammatory IL-10, IL-12p40 (Th1-related), and IL-4 (Th2-related) cytokines in RAW 264.7 cells, which have immunomodulatory effects [112].

Other probiotic EVs have also been shown to induce the differentiation of monocytic cells into the macrophage lineage. *L. plantarum* KCCM11179P (LEVs)-treated monocytes exhibit increased adherence, as well as increased expression of *CD14* and *ICAM-1* [64]. Moreover, LEVs-treated monocytes drive anti-inflammatory M2 macrophage polarization, as documented by increased expression of typical markers such as CD209, C-type lectin domain family 5-member A (CLEC5A), CD200 R, sphingosine kinase 1 (SPHK1), and class A scavenger receptor 1 (SRA1). The authors also revealed a significant increase in M2b macrophage-related chemokines, such as CCL1, CCL20, and CXCL2, which are related to tissue repair and anti-inflammatory activity [64].

EVs isolated from *B. vulgatus* mpk were shown to interact with naïve immune cells [55]. EV-induced semimaturation of CD11c^+^ bone marrow-derived DCs (BMDCs) manifested as increased, but still low, expression of the MHC-II, CD40, CD80, and CD86 receptors. BMDC maturation was also documented by the induced activation of both the TLR2 receptor and the CD14/TLR4/MD-2 receptor complex. Interestingly, EV-primed BMDCs did not respond to secondary stimulation with whole bacteria through changes in MHC-II expression or the secretion of TNF-α and IL-6, providing evidence for immune system silencing by *B. vulgatus* mpk EVs [55]. The induction of tolerant semimature DCs might play a significant role in preventing the development of inflammation-based diseases, including allergies.

In addition to macrophages and DCs, regulatory T cells (Tregs) play crucial roles in the development of allergies. Tregs balance Th1- and Th2-type immune responses, suppress allergen-specific IgE production and the activity of allergic inflammation effector cells [113]. Allergic diseases are associated with a low frequency and impaired function of Tregs [114], and Tregs have been suggested to be promising research target in allergy diagnosis and therapy [115]. DCs primed with *B. bifidum* LMG13195-derived EVs were shown to induce functional CD25^high^FOXP3^high^CD127^−/low^ Tregs from naïve T cells [58]. In contrast to isolated cytoplasmic proteins, envelope structures or inactivated bacteria are not as effective. EVs also induced the highest relative levels of IL-10 in CD4^+^ lymphocytes compared with the levels of the proinflammatory cytokines IFN-γ, TNF-α, and IL-17. These findings pave the way for the use of probiotic EVs as efficient adjuvants for immunotherapy.

Mast cells are the effector cells in allergies. Their FcεRI surface receptor binds allergens or IgE, which leads to cluster formation and cell activation. Its activation leads to the quick release of cytoplasmic granule-associated mediators such as histamine, heparin and other proteoglycans, cytokines, chemokines, growth factors, proteases, etc [116]. EVs obtained from *B. longum* KACC 91,563 interact with mast cells and induce their apoptosis [50], likely by the extracellular solute-binding protein (ESBP) which alleviates food allergy symptoms during OVA oral challenge [50]. These findings indicate that food allergies can be suppressed without activating the T-cell response but through the process of mast cell apoptosis induced by bacterial EVs.

Probiotic-derived EVs can modulate key immune mechanisms involved in allergy. By entering epithelial and immune cells, they influence signaling pathways such as NF-κB and MAPK, reducing the production of proinflammatory cytokines and promoting anti-inflammatory mediators like IL-10 and TGF-β. EV-primed dendritic cells acquire tolerogenic features and induce Tregs, which suppress Th2 responses and IgE production. They also drive macrophage polarization toward the anti-inflammatory M2 phenotype and can trigger mast cell apoptosis, directly dampening allergic reactions.

### In vivo studies

Since probiotic EVs show immunomodulatory properties in vitro, they have also been examined in more complicated models in vivo. The probiotic strain *E*. *coli* 083 (Colinfant Newborn) is a probiotic used in the clinic in high-risk infants to prevent nosocomial infections. However, long-term retrospective studies have shown that it also lowers the incidence of allergic sensitization later in life. Intranasal administration of *E*. *coli* O83 was shown to reduce allergic lung inflammation in mice through TLR4 activation [117]. Recently, it was shown that *E. coli* O83-derived EVs alleviate allergy symptoms in a mouse model of OVA-induced allergy [32]. Intranasal administration of EVs 30 min prior to each OVA treatment decreased the number of eosinophils in bronchoalveolar lavage (BAL) fluid after OVA challenge. Furthermore, EV treatment reduced allergen-specific IgA and IgE in BAL fluid. In vivo, OVA-restimulated lung cells produced significantly less IL-4, IL-5, IL-13, and IL-10 in the EV-treated group than in the sham-treated group. Similar results were observed in OVA-stimulated splenocytes from the EV-treated group [32]. Intranasal administration of *E. coli* O83-derived EVs to naïve mice induced time-dependent gene expression changes in the nasal-associated lymphoid tissue (NALT) and lungs [118]. After 2 h, BCL3, iNOS, and IL-6 were upregulated in the NALT, while NF-κB2, BCL3, SOD2, IL-6, and IL-10 increased in the lungs. EcO83-EVs also enhanced the recruitment of neutrophils, CD11b + dendritic cells, γδ T cells, and natural killer cells to the lungs. Rhodamine B tracking showed that lung macrophages primarily internalized the EVs, with uptake occurring independently of TLR4 signaling, as polymyxin B treatment did not inhibit this process.

Oral administration of *L. paracasei-*derived EVs alleviated airway resistance and inflammation in C57BL/6 mice with neutrophilic asthma induced by LPS [119]. Compared with asthmatic control mice. EV-treated asthmatic mice presented significant decreases in airway hyperresponsiveness, neutrophil counts, and the levels of CXCL1 and IL-17 in the BAL fluid. The suppressive effects of EVs were even stronger than those of the anti-asthmatic drug dexamethasone. Moreover, EVs reduced the ratio of CD4^+^RORγt^+^ Th17 cells to FOXP3^+^ Treg cells in the lungs. Sim et al. suggested that the effect comes from cargo molecules, such as dodecanoic acid, palmitoleic acid, and D-(-)-tagatose, which significantly inhibited JNK phosphorylation and IL-8 production in human airway epithelial cells pretreated with LPS, similar to EVs [119]. Similar results were obtained for *Lactococcus lactis* EVs [120]. Moreover, these probiotic EVs decreased eosinophil numbers and decreased the levels of IL-5 and IL-13 in BAL fluid. Interestingly, EVs increased the level of IFN-γ in BAL fluid and may thus provide beneficial Th1 immunomodulatory signals. The EV group also presented lower expression of the Th2-related transcription factor GATA-3 and reduced phosphorylation of STAT6 in lung tissue. The authors suggested that since EVs were unable to demonstrate their antiallergic potential in the IL-12 neutralization study, the health-promoting effect may be related to the presence of specific cargo that induces IL-12 production [120]. Oral administration of EVs isolated from the probiotic EcN and the commensal EcoR12 to neonatal rats stimulated the innate and adaptative immune systems [121]. The administration of EVs isolated from both *E. coli* strains resulted in elevated total levels of plasma IgG, IgM, and IgA and promoted a shift toward the Th1 response. EVs also increased the proportions of cytotoxic lymphocytes (TCRαβ^+^ 9 of 19 CD8^+^ NK−) and natural killer T cells (TCRαβ^+^ CD8^+^ NK^+^), which are important for fighting intracellular pathogens. Additionally, the administration of EcN-derived EVs led to a reduction in IL-12 expression and downregulation of the proinflammatory enzyme COX-2. Both types of EVs increased OCLDN expression, which is involved in intestinal barrier integrity [121].

## The role of probiotic-derived EVs in gut microbiota modulation

The development and maintenance of a healthy microbiome is a key to human well-being, as bacteria contribute to multiple physiological functions. The healthy human microbiome promotes intestinal epithelial and mucosal integrity, produces essential vitamins, and, more importantly from an allergy point of view, has a potent immunomodulatory role. An imbalance or maladaptation of the microbiota, defined as dysbiosis, is recognized as an important factor in the development of allergies. Research has demonstrated that restoring the gastrointestinal microbial balance may have a therapeutic effect on allergies [122, 123]. Oral administration of a combination of *L. acidophilus* NCFMTM and *B. lactis* Bl-04 positively affects markers of respiratory allergy, particularly in mucous membranes [124] and resulted in a reduction in allergy-related symptoms in the nose. To date, there are no reports on the effects of EVs on the microbiota connected with allergies; nevertheless, there might be an indirect impact of EVs on the microbiota related to the levels of induced proinflammatory cytokines or influencing the oxidative stress in the gut [125]. Pathogenic *Proteobacteria*, such as *Helicobacter*,* Escherichia*, and *Klebsiella*, were positively associated with the induction of proinflammatory cytokines. In contrast, beneficial microbes such as *Muribaculaceae*,* Bacteroides*,* Akkermansia*, and *Bifidobacterium* were negatively correlated with the induction of proinflammatory cytokines. EVs from *L. plantarum* Q7 CP019712-16, when administered by oral gavage to mice with DSS-induced colitis, lowered the levels of the proinflammatory cytokines IL-6, IL-1β, and TNF-α and increased the IL-2 level almost to physiological values, which had an impact on the gut microbiota composition. The presence of EVs resulted in increased quantities of *Akkermansia*,* Bifidobacteria*,* Muribaculaceae*, and *Lactobacillus*, which are linked with reduced allergy symptoms [28, 126–128]. Conversely, EVs decreased the abundance of *Proteobacteria*,* Deferribacteres*, and *Epsilonbacteraeota* [65]. Moreover, metagenomic sequencing revealed that EVs derived from *Clostridium butyricum* have a rebalancing effect on the microbiota of mice with experimental colitis by decreasing the presence of the bacterial pathogens *E. coli* and *Shigella flexneri*. Furthermore, the relative abundances of beneficial species such as *B. pseudolongum*,* A. glycaniphila*, and *A. muciniphila* were greater in the EV-treated group than in the control DSS group [60]. There are also some first reports of a direct antibacterial effect of probiotic EVs. For example, cattle milk-derived EVs that have undergone pasteurization demonstrate a dose-dependent ability to inhibit the growth of *Staphylococcus aureus*, leading to an extension of the lag and generation times [129]. Lowering the amounts of proinflammatory strains might play a significant role in the development or treatment of different types of allergies.

## Conclusions

**Fig. 2:**
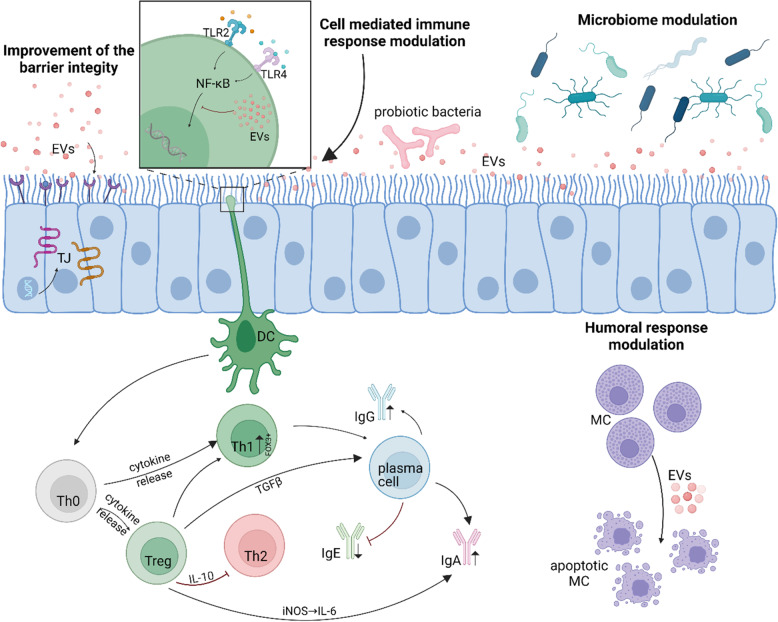
Proposed mechanisms of action of probiotic EVs. EVs, extracellular vesicles; TJ, tight junctions; TLR, toll-like receptors; DC, dendritic cells; MC, mast cells; iNOS, nitric oxide synthase; Th, T helper cells; Treg, T regulatory cells.

Probiotic EVs are postbiotics gaining increasing attention for their immunomodulatory properties. While studies confirm their efficacy in regulating immune responses (Table 1), their application in allergy models remains limited. This review explores the mechanisms by which probiotic EVs may contribute to anti-allergic therapy (Fig. 2). We propose four primary mechanisms through which probiotic EVs could exert their effects in the context of allergy: (1) enhancement of barrier integrity; (2) modulation of cell-mediated immune responses; (3) regulation of humoral responses; and (4) alteration of microbiota composition.

Tight junctions (TJs) are key for maintaining epithelial barrier integrity; their dysfunction is linked to allergic diseases like asthma and atopic dermatitis [94], as barrier damage increases allergen exposure and hypersensitivity risk [130, 131]. A strong barrier lessens cytokine release and allergic symptoms. Probiotic EVs modulate TJs such as ZO-1, occludin, and claudins, strengthening gut, lung, and skin barriers and improving resistance to allergens.

Regulation of cell-mediated immunity is essential for allergy prevention. Treg cells suppress excessive responses to allergens, promoting tolerance [132]. Allergy-prone individuals often have a Th2-dominant response, which increases inflammation. Targeting modulation of key Th2 cytokines, including IL-4, IL-5, and IL-13, can decrease IgE synthesis and eosinophil activation, leading to attenuation of chronic inflammatory responses [133]. Additionally, cytokines like IL-22, strengthen epithelial barriers, preventing allergen penetration. Early regulation of T cell responses is vital to halt the progression of allergies into severe conditions, such as persistent asthma [132]. Probiotic EVs can shift immune responses toward Th1/Treg by influencing DCs, increasing IL-10, IFN-ɤ, or TGF-β, and suppressing Th2 cytokines. This suggests that EVs help promote immune tolerance and reduce allergic inflammation.

Mast cells drive allergic reactions by releasing histamine [134]. Inducing their apoptosis lowers histamine levels and eases symptoms, especially in chronic allergies like asthma and eczema [135]. Research shows probiotic EVs trigger mast cell apoptosis via ESBP [50], reducing histamine release and long-term mast cell buildup, indicating EV-based therapies may help control mast cell-driven conditions.like asthma, atopic dermatitis, and food allergies.

A balanced microbiome promotes Treg cell development and limits allergies. Dysbiosis is linked to asthma and eczema, often due to lower levels of beneficial bacteria like *Bifidobacterium* [136]. Gut bacteria produce SCFAs that strengthen barriers and reduce allergen penetration [137]. Additionally, microbiome affects B cell activity and IgE production, further regulating allergies. Early-life microbiome modulation through probiotics and dietary strategies can lower the risk of allergic diseases, and a healthy microbiome enhances the effectiveness of immunotherapy and anti-inflammatory treatments [138]. EVs from *L. plantarum* and *C. butyricum* boost beneficial microbiota and lower inflammatory pathogens [60], showing EVs can modulate the microbial ecosystem and help prevent allergies and inflammatory diseases.

## Future directions

Probiotic EVs present a promising alternative to live bacteria in the prevention and treatment of allergies, particularly for immunocompromised individuals, due to their reduced risk of adverse effects. These EVs are capable of delivering a diverse array of biomolecules to host cells and can be engineered for targeted drug delivery, thereby improving both stability and efficacy in allergy immunotherapy. They interact with host cells through multiple pathways, with certain EVs possessing adhesion molecules that enable specific receptor targeting. By enhancing the pharmacodynamics and pharmacokinetics of their content, EVs emerge as superior candidates for allergy treatment compared to traditional bacterial therapies.

Allergen-specific immunotherapy (AIT) is designed to desensitize the immune system to specific allergens, providing enduring relief from allergic symptoms [133]. EVs can function as effective carriers in AIT, owing to their biocompatibility and innate capacity to interact with the immune system [139]. Through engagement with various immune cell types, EVs promote a balanced immune response, thereby potentially mitigating hypersensitivity to allergens. When combined with conventional AIT, probiotic EVs may amplify therapeutic effects and lessen side effects, acting as adjuvants to enhance immune tolerance toward allergens [140]. To fully realize the potential of probiotic EVs in allergy therapy, several modifications can be employed. One approach involves surface engineering, wherein EVs are modified with specific ligands- including peptides or antibodies- to facilitate targeted delivery and improve therapeutic outcomes while minimizing off-target effects [141]. Another strategy encompasses cargo loading with allergens, wherein specific allergens are incorporated into EVs to enable controlled exposure, thus desensitizing the immune system and reducing allergic reactions. Techniques such as incubation, electroporation, sonication, and extrusion can be utilized to achieve efficient allergen loading [142]. Furthermore, enhancing the stability and circulation time of EVs is vital; PEGylation, the process of attaching polyethylene glycol chains, serves as an effective technique to improve EVs stability in the bloodstream, prolong circulation time, and enhance therapeutic efficacy [143]. Overall, EVs improve the pharmacodynamics and pharmacokinetics of their contents, making them superior candidates for allergy treatment compared to traditional bacteria.

Despite their potential, several challenges must be addressed before probiotic EVs can be applied clinically. Standardization of EV production, purification, and characterization is crucial, as variations in isolation techniques can alter EV composition and functionality. Key parameters, including bacterial culture conditions and isolation methods, need to be clearly defined. Inconsistent EV purification can affect bioactivity by losing essential surface proteins or molecular cargo during processing. Currently, the International Society for Extracellular Vesicles (ISEV) has established minimal guidelines (MISEV) for EV isolation and functional analysis [144], while tools like EV-TRACK [145] help assess the accuracy of purification and characterization methods. However, more efficient and scalable isolation methods are needed, particularly for obtaining EVs from natural sources such as blood or the intestinal lumen, where current techniques yield low quantities and require extensive purification steps [146]. Additionally, universal biomarkers for bacterial EVs remain elusive. While LPS and LTA have been proposed as biomarkers, their structural variability between bacterial strains raises concerns about their reliability [147]. Moreover, the biological properties of EVs produced in vitro may differ significantly from those generated in vivo, as factors such as culture medium composition, pH, salinity, and temperature can dramatically alter EV content and function.

Although clinical applications remain largely unexplored, probiotic EVs offer an attractive strategy for allergy management that extends beyond traditional antihistamines and immunosuppressants. Future research should focus on optimizing EV production, standardizing methodologies, and exploring their interactions with host immune cells and microbiota. By addressing these challenges, probiotic EVs could pave the way for next-generation allergy therapeutics with enhanced precision and safety.

## Data Availability

No datasets were generated or analysed during the current study.

## Abbreviations

AD: Atopic dermatitis
AIT: Allergen-specific immunotherapy
AMPK: AMP-activated protein kinase
APC: Antigen-presenting cell
AR: Allergic rhinitis
BAL: Bronchoalveolar lavage
BMP: Bone morphogenetic protein
CLDN: Claudin
COX: 2-Cyclooxygenase-2
DC: Dendritic cell
DAMP: Damage-associated molecular pattern
DSS: Dextran sulfate sodium
EGFR: Epidermal growth factor receptor
ER: Endoplasmic reticulum
ESBP: Extracellular solute-binding protein
EV: Extracellular vesicle
FA: Food allergy
FOS: FBJ murine osteosarcoma viral oncogene homolog
FoxP3: Forkhead box P3
GATA: 3-GATA binding protein 3
ICAM: 1-Intercellular adhesion molecule 1
IDO: Indoleamine-pyrrole 2,3-dioxygenase
IFN-γ: Interferon gamma
IgA/IgE/IgG/IgM: Immunoglobulin A / E / G / M
IL-1, IL-2, ..., IL-33: Interleukin 1, 2, ..., 33
iNOS: Inducible nitric oxide synthase
ISBP: Iron-sulfur cluster binding protein (context-dependent)
JNK: c-Jun N-terminal kinase
LPS: Lipopolysaccharide
LTB: Lymphotoxin beta
LTA: Lipoteichoic acid
MAPK: Mitogen-activated protein kinase
MC: Mast cell
MISEV: Minimal Information for Studies of Extracellular Vesicles
MPO: Myeloperoxidase
NALT: Nasal-associated lymphoid tissue
NF-κB: Nuclear factor kappa-light-chain-enhancer of activated B cells
NO: Nitric oxide
NOD: Nucleotide-binding oligomerization domain
OVA: Ovalbumin
PAR: Protease-activated receptor
PEGylation: Polyethylene glycol modification
PG: Peptidoglycan
PRR: Pattern recognition receptor
RIG: I-Retinoic acid-inducible gene I
RT: qPCR-Reverse transcription quantitative polymerase chain reaction
SCFA: Short-chain fatty acid
STAT6: Signal transducer and activator of transcription 6
TGF-β: Transforming growth factor beta
Th1/Th2/Th17: T helper cell types 1 / 2 / 17
TJ: Tight junction
TLR: Toll-like receptor
TNF-α / TNF-β: Tumor necrosis factor alpha / beta
Treg: Regulatory T cell
TSLP: Thymic stromal lymphopoietin
ZO-1: Zonula occluden-1

## References

[1] Passani MB, Giannoni P, Bucherelli C, Baldi E, Blandina P. Histamine in the brain: beyond sleep and memory. Biochem Pharmacol. 2007;73(8):1113–22. 17241615 10.1016/j.bcp.2006.12.002

[2] Manea I, Ailenei E, Deleanu D. Overview of food allergy diagnosis. Med Pharm Rep. 2016;89(1):5–10. 10.15386/cjmed-513 PMC4777468 27004019

[3] Agache I, Annesi-Maesano I, Bonertz A, Branca F, Cant A, Fras Z, et al. Prioritizing research challenges and funding for allergy and asthma and the need for translational research—The European strategic forum on allergic diseases. Allergy. 2019;74(11):2064–76. 31070805 10.1111/all.13856

[4] Ridolo E, Incorvaia C, Pucciarini F, Makri E, Paoletti G, Canonica GW. Current treatment strategies for seasonal allergic rhinitis: where are we heading? Clin Mol Allergy. 2022;20(1):9. 35948975 10.1186/s12948-022-00176-x PMC9367100

[5] Jakubczyk D, Górska S. Impact of probiotic bacteria on respiratory allergy disorders. Front Microbiol. 2021. 10.3389/fmicb.2021.688137. 34234762 10.3389/fmicb.2021.688137 PMC8256161

[6] Marsland BJ, Trompette A, Gollwitzer ES. The gut-lung axis in respiratory disease. Ann Am Thorac Soc. 2015;12(Suppl 2):S150-156. 26595731 10.1513/AnnalsATS.201503-133AW

[7] Dang AT, Marsland BJ. Microbes, metabolites, and the gut–lung axis. Mucosal Immunol. 2019;12(4):843–50. 30976087 10.1038/s41385-019-0160-6

[8] Chen JC, Tsai CC, Hsieh CC, Lan A, Huang CC, Leu SF. Multispecies probiotics combination prevents ovalbumin-induced airway hyperreactivity in mice. Allergol Immunopathol (Madr). 2018;46(4):354–60. 29739682 10.1016/j.aller.2018.02.001

[9] Spacova I, Van Beeck W, Seys S, Devos F, Vanoirbeek J, Vanderleyden J, et al. *Lactobacillus rhamnosus* probiotic prevents airway function deterioration and promotes gut microbiome resilience in a murine asthma model. Gut Microbes. 2020;11(6):1729–44. 32522072 10.1080/19490976.2020.1766345 PMC7524350

[10] Enaud R, Prevel R, Ciarlo E, Beaufils F, Wieërs G, Guery B, et al. The gut-lung axis in health and respiratory diseases: a place for inter-organ and inter-kingdom crosstalks. Front Cell Infect Microbiol. 2020;10:9. 32140452 10.3389/fcimb.2020.00009 PMC7042389

[11] Spacova I, Ceuppens JL, Seys SF, Petrova MI, Lebeer S. Probiotics against airway allergy: host factors to consider. Dis Model Mech. 2018;11(7):dmm034314. 30037806 10.1242/dmm.034314 PMC6078401

[12] Luo C, Peng S, Li M, Ao X, Liu Z. The efficacy and safety of probiotics for allergic rhinitis: a systematic review and meta-analysis. Front Immunol. 2022;13:848279. 35663980 10.3389/fimmu.2022.848279 PMC9161695

[13] Kim K, Lee E, Kim M, Lee KS, Sol IS, Min TK et al. Therapeutic effectiveness of probiotics for atopic dermatitis: A systematic review and meta-analysis of randomized controlled trials with subgroup analysis. Asian Pac J Allergy Immunol. 2023. 10.12932/AP-280323-1576 37578483

[14] Balan D, Baral T, Manu MK, Mohapatra AK, Miraj SS. Efficacy of probiotics as adjuvant therapy in bronchial asthma: a systematic review and meta-analysis. Allergy Asthma Clin Immunol. 2024;20(1):60. 39563347 10.1186/s13223-024-00922-7 PMC11575415

[15] Farahmandi K, Mohr AE, McFarland LV. Effects of probiotics on allergic rhinitis: a systematic review and meta-analysis of randomized clinical trials. Am J Rhinol Allergy. 2022;36(4):440–50. 35099301 10.1177/19458924211073550

[16] Wang Y, Wang B, Sun S, Wang Z. Mapping the relationship between atopic dermatitis and gut microbiota: a bibliometric analysis, 2014–2023. Front Microbiol [Internet]. 2024 Sep 4 [cited 2025 Oct 14];15. Available from: https://www.frontiersin.org/journals/microbiology/articles/10.3389/fmicb.2024.1400657/full 10.3389/fmicb.2024.1400657 PMC11408322 39296293

[17] Lee SY, Lee E, Park YM, Hong SJ. Microbiome in the gut-skin axis in atopic dermatitis. Allergy Asthma Immunol Res. 2018;10(4):354–62. 29949831 10.4168/aair.2018.10.4.354 PMC6021588

[18] Leo S, Cetiner OF, Pittet LF, Messina NL, Jakob W, Falquet L et al. The association between the composition of the early-life intestinal microbiome and eczema in the first year of life. Front Microbiomes [Internet]. 2023 Mar 16 [cited 2025 Oct 14];2. Available from: https://www.frontiersin.org/journals/microbiomes/articles/10.3389/frmbi.2023.1147082/full 10.3389/frmbi.2023.1147082 PMC12993559 41853385

[19] Liu Y, Du X, Zhai S, Tang X, Liu C, Li W. Gut microbiota and atopic dermatitis in children: a scoping review. BMC Pediatr. 2022;22(1):323. 35655175 10.1186/s12887-022-03390-3 PMC9161518

[20] Díez-Madueño K, de la Cueva Dobao P, Torres-Rojas I, Fernández-Gosende M, Hidalgo-Cantabrana C, Coto-Segura P. Gut dysbiosis and adult atopic dermatitis: a systematic review. J Clin Med. 2024;14(1):19. 39797102 10.3390/jcm14010019 PMC11721037

[21] Avcin SL, Pokorn M, Kitanovski L, Premru MM, Jazbec J. Bifidobacterium breve Sepsis in Child with High-Risk Acute Lymphoblastic Leukemia - Volume 21, Number 9—September 2015 - Emerging Infectious Diseases journal - CDC. 2022 Feb 3 [cited 2022 Feb 3]; Available from: https://wwwnc.cdc.gov/eid/article/21/9/15-0097_article 10.3201/eid2109.150097 PMC4550169 26291071

[22] Esaiassen E, Hjerde E, Cavanagh JP, Simonsen GS, Klingenberg C. *Bifidobacterium* bacteremia: clinical characteristics and a genomic approach to assess pathogenicity. J Clin Microbiol. 2017. 10.1128/JCM.00150-17. 28490487 10.1128/JCM.00150-17 PMC5483926

[23] Marteau P, Shanahan F. Basic aspects and Pharmacology of probiotics: an overview of pharmacokinetics, mechanisms of action and side-effects. Best Pract Res Clin Gastroenterol. 2003;17(5):725–40. 14507584 10.1016/s1521-6918(03)00055-6

[24] Yelin I, Flett KB, Merakou C, Mehrotra P, Stam J, Snesrud E, et al. Genomic and epidemiological evidence of bacterial transmission from probiotic capsule to blood in ICU patients. Nat Med. 2019;25(11):1728–32. 31700189 10.1038/s41591-019-0626-9 PMC6980696

[25] Vassilopoulou E, Comotti A, Douladiris N, Konstantinou GΝ, Zuberbier T, Alberti I, et al. A systematic review and meta-analysis of nutritional and dietary interventions in randomized controlled trials for skin symptoms in children with atopic dermatitis and without food allergy: an EAACI task force report. Allergy. 2024. 10.1111/all.16160. 38783644 10.1111/all.16160

[26] Thoda C, Touraki M. Immunomodulatory properties of probiotics and their derived bioactive compounds. Appl Sci. 2023;13(8):4726.

[27] Ivory K, Chambers SJ, Pin C, Prieto E, Arqués JL, Nicoletti C. Oral delivery of *Lactobacillus casei* Shirota modifies allergen-induced immune responses in allergic rhinitis. Clin Exp Allergy. 2008;38(8):1282–9. 18510694 10.1111/j.1365-2222.2008.03025.x

[28] Pyclik MJ, Srutkova D, Razim A, Hermanova P, Svabova T, Pacyga K, et al. Viability status-dependent effect of *bifidobacterium longum* ssp. *longum* CCM 7952 on prevention of allergic inflammation in mouse model. Front Immunol. 2021;12:707728. 34354710 10.3389/fimmu.2021.707728 PMC8329652

[29] Pacyga-Prus K, Jakubczyk D, Sandström C, Šrůtková D, Pyclik MJ, Leszczyńska K, et al. Polysaccharide BAP1 of *bifidobacterium adolescentis* CCDM 368 is a biologically active molecule with immunomodulatory properties. Carbohydr Polym. 2023;315:120980. 37230638 10.1016/j.carbpol.2023.120980

[30] Pacyga-Prus K, Sandström C, Šrůtková D, Schwarzer M, Górska S. Phosphorylation-dependent immunomodulatory properties of B.PAT polysaccharide isolated from *bifidobacterium animalis* ssp. *animalis* CCDM 218. Carbohydr Polym. 2024;344:122518. 39218543 10.1016/j.carbpol.2024.122518

[31] Srutkova D, Kozakova H, Novotna T, Gorska S, Hermanova PP, Hudcovic T, et al. Exopolysaccharide from Lacticaseibacillus rhamnosus induces IgA production in airways and alleviates allergic airway inflammation in mouse model. Eur J Immunol. 2023;53(7):e2250135. 37177812 10.1002/eji.202250135

[32] Schmid AM, Razim A, Wysmołek M, Kerekes D, Haunstetter M, Kohl P, et al. Extracellular vesicles of the probiotic bacteria *E. coli* O83 activate innate immunity and prevent allergy in mice. Cell Commun Signal. 2023;21(1):297. 37864211 10.1186/s12964-023-01329-4 PMC10588034

[33] Knox KW, Vesk M, Work E. Relation between excreted lipopolysaccharide complexes and surface structures of a Lysine-limited culture of *Escherichia coli*. J Bacteriol. 1966;92(4):1206–17. 4959044 10.1128/jb.92.4.1206-1217.1966 PMC276396

[34] Kim DK, Kang B, Kim OY, Choi DS, Lee J, Kim SR, et al. EVpedia: an integrated database of high-throughput data for systemic analyses of extracellular vesicles. J Extracell Vesicles. 2013. 10.3402/jev.v2i0.20384. 24009897 10.3402/jev.v2i0.20384 PMC3760654

[35] Bonnington KE, Kuehn MJ. Protein selection and export via outer membrane vesicles. Biochim Biophys Acta. 2014;1843(8):1612–9. 24370777 10.1016/j.bbamcr.2013.12.011 PMC4317292

[36] Kuehn MJ, Kesty NC. Bacterial outer membrane vesicles and the host–pathogen interaction. Genes Dev. 2005;19(22):2645–55. 16291643 10.1101/gad.1299905

[37] Chowdhury C, Jagannadham MV. Virulence factors are released in association with outer membrane vesicles of *Pseudomonas syringae* pv. tomato T1 during normal growth. Biochimica et Biophysica Acta (BBA). 2013;1834(1):231–9. 23043909 10.1016/j.bbapap.2012.09.015

[38] Dorward DW, Garon CF. DNA is packaged within membrane-derived vesicles of Gram-negative but not Gram-positive bacteria. Appl Environ Microbiol. 1990;56(6):1960–2. 16348232 10.1128/aem.56.6.1960-1962.1990 PMC184538

[39] Kim JH, Lee J, Park J, Gho YS. Gram-negative and Gram-positive bacterial extracellular vesicles. Semin Cell Dev Biol. 2015;40:97–104. 25704309 10.1016/j.semcdb.2015.02.006

[40] Bäuerl C, Coll-Marqués JM, Tarazona-González C, Pérez-Martínez G. *Lactobacillus casei* extracellular vesicles stimulate EGFR pathway likely due to the presence of proteins P40 and P75 bound to their surface. Sci Rep. 2020;10(1):19237. 33159116 10.1038/s41598-020-75930-9 PMC7648624

[41] Schwechheimer C, Kuehn MJ. Outer-membrane vesicles from gram-negative bacteria: biogenesis and functions. Nat Rev Microbiol. 2015;13(10):605–19. 26373371 10.1038/nrmicro3525 PMC5308417

[42] Briaud P, Carroll RK. Extracellular vesicle biogenesis and functions in Gram-Positive bacteria. Infect Immun. 2020;88(12). 10.1128/iai.00433-20. 10.1128/IAI.00433-20 PMC7671900 32989035

[43] Hosseini-Giv N, Basas A, Hicks C, El-Omar E, El-Assaad F, Hosseini-Beheshti E. Bacterial extracellular vesicles and their novel therapeutic applications in health and cancer. Front Cell Infect Microbiol. 2022;12:962216. 36439225 10.3389/fcimb.2022.962216 PMC9691856

[44] Zhang X, Wang Y, E Q, Naveed M, Wang X, Liu Y, et al. The biological activity and potential of probiotics-derived extracellular vesicles as postbiotics in modulating microbiota-host communication. J Nanobiotechnol. 2025;23(1):349. 10.1186/s12951-025-03435-6 PMC12082936 40380331

[45] Sartorio MG, Pardue EJ, Feldman MF, Haurat MF. Bacterial outer membrane vesicles: from discovery to applications. Annu Rev Microbiol. 2021;75(1):609–30. 34351789 10.1146/annurev-micro-052821-031444 PMC8500939

[46] De Langhe N, Van Dorpe S, Guilbert N, Vander Cruyssen A, Roux Q, Deville S, et al. Mapping bacterial extracellular vesicle research: insights, best practices and knowledge gaps. Nat Commun. 2024;15(1):9410. 39482295 10.1038/s41467-024-53279-1 PMC11528011

[47] Bielig H, Dongre M, Zurek B, Wai SN, Kufer TA. A role for quorum sensing in regulating innate immune responses mediated by *Vibrio cholerae* outer membrane vesicles (OMVs). Gut Microbes. 2011;2(5):274–9. 22067940 10.4161/gmic.2.5.18091

[48] Ewaschuk JB, Diaz H, Meddings L, Diederichs B, Dmytrash A, Backer J, et al. Secreted bioactive factors from bifidobacterium infantis enhance epithelial cell barrier function. Am J Physiology-Gastrointestinal Liver Physiol. 2008;295(5):G1025–34. 10.1152/ajpgi.90227.2008 18787064

[49] Fábrega MJ, Rodríguez-Nogales A, Garrido-Mesa J, Algieri F, Badía J, Giménez R, et al. Intestinal anti-inflammatory effects of outer membrane vesicles from *Escherichia coli* Nissle 1917 in DSS-experimental colitis in mice. Front Microbiol. 2017. 10.3389/fmicb.2017.01274. 28744268 10.3389/fmicb.2017.01274 PMC5504144

[50] Kim JH, Jeun EJ, Hong CP, Kim SH, Jang MS, Lee EJ, et al. Extracellular vesicle–derived protein from bifidobacterium longum alleviates food allergy through mast cell suppression. J Allergy Clin Immunol. 2016;137(2):507–e5168. 26433560 10.1016/j.jaci.2015.08.016

[51] Behzadi E, Mahmoodzadeh Hosseini H, Imani Fooladi AA. The inhibitory impacts of *Lactobacillus rhamnosus* GG-derived extracellular vesicles on the growth of hepatic cancer cells. Microb Pathog. 2017;110:1–6. 28634130 10.1016/j.micpath.2017.06.016

[52] Liu Y, Defourny KAY, Smid EJ, Abee T. Gram-Positive Bacterial Extracellular Vesicles and Their Impact on Health and Disease. Frontiers in Microbiology [Internet]. 2018 [cited 2022 Feb 2];9. Available from: https://www.frontiersin.org/article/10.3389/fmicb.2018.01502 10.3389/fmicb.2018.01502 PMC6046439 30038605

[53] Alvarez CS, Badia J, Bosch M, Giménez R, Baldomà L. Outer Membrane Vesicles and Soluble Factors Released by Probiotic Escherichia coli Nissle 1917 and Commensal ECOR63 Enhance Barrier Function by Regulating Expression of Tight Junction Proteins in Intestinal Epithelial Cells. Frontiers in Microbiology [Internet]. 2016 [cited 2022 May 1];7. Available from: https://www.frontiersin.org/article/10.3389/fmicb.2016.01981 10.3389/fmicb.2016.01981 PMC5156689 28018313

[54] Ashrafian F, Behrouzi A, Shahriary A, Badi SA, Davari M, Khatami S, et al. Comparative study of effect of Akkermansia muciniphila and its extracellular vesicles on toll-like receptors and tight junction. Gastroenterol Hepatol Bed Bench. 2019;12(2):163–8. 31191842 PMC6536015

[55] Maerz JK, Steimle A, Lange A, Bender A, Fehrenbacher B, Frick JS. Outer membrane vesicles blebbing contributes to B. vulgatus mpk-mediated immune response silencing. Gut Microbes. 2018;9(1):1–12. 28686482 10.1080/19490976.2017.1344810 PMC5914909

[56] Zakharzhevskaya NB, Vanyushkina AA, Altukhov IA, Shavarda AL, Butenko IO, Rakitina DV, et al. Outer membrane vesicles secreted by pathogenic and nonpathogenic *Bacteroides fragilis* represent different metabolic activities. Sci Rep. 2017;7(1):5008. 28694488 10.1038/s41598-017-05264-6 PMC5503946

[57] Zheng C, Zhong Y, Xie J, Wang Z, Zhang W, Pi Y, et al. *Bacteroides acidifaciens* and its derived extracellular vesicles improve DSS-induced colitis. Front Microbiol. 2023;14:1304232. 38098663 10.3389/fmicb.2023.1304232 PMC10720640

[58] López P, González-Rodríguez I, Sánchez B, Gueimonde M, Margolles A, Suárez A. Treg-inducing membrane vesicles from *bifidobacterium bifidum* LMG13195 as potential adjuvants in immunotherapy. Vaccine. 2012;30(5):825–9. 22172507 10.1016/j.vaccine.2011.11.115

[59] Hassani S, Sotoodehnejadnematalahi F, Fateh A, Siadat SD. Evaluation of association between *bifidobacterium bifidum* derived extracellular vesicles and intestinal epithelium tight junction proteins through Notch-1 and AhR activation in Caco-2 cell line. Mol Genet Microbiol Virol. 2021;36(1):S1-6.

[60] Ma L, Lyu W, Song Y, Chen K, Lv L, Yang H, et al. Anti-Inflammatory effect of clostridium butyricum-Derived extracellular vesicles in ulcerative colitis: impact on host MicroRNAs expressions and gut Microbiome profiles. Mol Nutr Food Res. 2023;67(13):e2200884. 37183784 10.1002/mnfr.202200884

[61] Seo MK, Park EJ, Ko SY, Choi EW, Kim S. Therapeutic effects of kefir grain *Lactobacillus*-derived extracellular vesicles in mice with 2,4,6-trinitrobenzene sulfonic acid-induced inflammatory bowel disease. J Dairy Sci. 2018;101(10):8662–71. 30100498 10.3168/jds.2018-15014

[62] Fan J, Zhang Y, Zuo M, Ding S, Li J, Feng S, et al. Novel mechanism by which extracellular vesicles derived from *Lactobacillus murinus* alleviates deoxynivalenol-induced intestinal barrier disruption. Environ Int. 2024;185:108525. 38408410 10.1016/j.envint.2024.108525

[63] Champagne-Jorgensen K, Jose TA, Stanisz AM, Mian MF, Hynes AP, Bienenstock J. Bacterial membrane vesicles and phages in blood after consumption of *Lacticaseibacillus rhamnosus* JB-1. Gut Microbes. 2021;13(1):1993583. 34747333 10.1080/19490976.2021.1993583 PMC8583084

[64] Kim W, Lee EJ, Bae IH, Myoung K, Kim ST, Park PJ, et al. *Lactobacillus plantarum*-derived extracellular vesicles induce anti-inflammatory M2 macrophage polarization in vitro. J Extracell Vesicles. 2020;9(1):1793514. 32944181 10.1080/20013078.2020.1793514 PMC7480564

[65] Hao H, Zhang X, Tong L, Liu Q, Liang X, Bu Y, et al. Effect of extracellular vesicles derived from *Lactobacillus plantarum* Q7 on gut microbiota and ulcerative colitis in mice. Front Immunol. 2021;12:777147. 34925349 10.3389/fimmu.2021.777147 PMC8674835

[66] Yamasaki-Yashiki S, Kawashima F, Saika A, Hosomi R, Kunisawa J, Katakura Y. RNA-based anti-inflammatory effects of membrane vesicles derived from *Lactiplantibacillus plantarum*. Foods. 2024;13(6):967. 38540957 10.3390/foods13060967 PMC10969829

[67] Choi JH, Moon CM, Shin TS, Kim EK, McDowell A, Jo MK, et al. *Lactobacillus paracasei*-derived extracellular vesicles attenuate the intestinal inflammatory response by augmenting the Endoplasmic reticulum stress pathway. Exp Mol Med. 2020;52(3):423–37. 32123288 10.1038/s12276-019-0359-3 PMC7156483

[68] Yamasaki-Yashiki S, Miyoshi Y, Nakayama T, Kunisawa J, Katakura Y. IgA-enhancing effects of membrane vesicles derived from *Lactobacillus sakei* subsp. *sakei* NBRC15893. Biosci Microbiota Food Health. 2019;38(1):23–9. 30705799 10.12938/bmfh.18-015 PMC6343049

[69] Hu R, Lin H, Wang M, Zhao Y, Liu H, Min Y, et al. *Lactobacillus reuteri*-derived extracellular vesicles maintain intestinal immune homeostasis against lipopolysaccharide-induced inflammatory responses in broilers. J Anim Sci Biotechnol. 2021;12(1):25. 33593426 10.1186/s40104-020-00532-4 PMC7888134

[70] Kang EA, Choi HI, Hong SW, Kang S, Jegal HY, Choi EW, et al. Extracellular vesicles derived from Kefir grain *Lactobacillus* ameliorate intestinal inflammation via regulation of proinflammatory pathway and tight junction integrity. Biomedicines. 2020;8(11):E522. 10.3390/biomedicines8110522 PMC7709018 33233771

[71] Vargoorani ME, Modarressi MH, Vaziri F, Motevaseli E, Siadat SD. Stimulatory effects of *Lactobacillus casei* derived extracellular vesicles on toll-like receptor 9 gene expression and cytokine profile in human intestinal epithelial cells. J Diabetes Metab Disord. 2020;19(1):223–31. 32550171 10.1007/s40200-020-00495-3 PMC7270429

[72] Moosavi SM, Akhavan Sepahi A, Mousavi SF, Vaziri F, Siadat SD. The effect of *Faecalibacterium prausnitzii* and its extracellular vesicles on the permeability of intestinal epithelial cells and expression of PPARs and ANGPTL4 in the Caco-2 cell culture model. J Diabetes Metab Disord. 2020;19(2):1061–9. 33520823 10.1007/s40200-020-00605-1 PMC7843710

[73] Rodovalho V, de Luz R, da Rabah BSR, do Carmo H, Folador FLR, Nicolas EL et al. A,. Extracellular Vesicles Produced by the Probiotic Propionibacterium freudenreichii CIRM-BIA 129 Mitigate Inflammation by Modulating the NF-κB Pathway. Frontiers in Microbiology [Internet]. 2020 [cited 2022 Feb 2];11. Available from: https://www.frontiersin.org/article/10.3389/fmicb.2020.01544 10.3389/fmicb.2020.01544 PMC7359729 32733422

[74] Jahromi LP, Fuhrmann G. Bacterial extracellular vesicles: understanding biology promotes applications as nanopharmaceuticals. Adv Drug Deliv Rev. 2021;173:125–40. 33774113 10.1016/j.addr.2021.03.012

[75] Willart W. Hammad. Alarming dendritic cells for allergic sensitization. Allergology international: official journal of the Japanese Society of Allergology [Internet]. 2010 Jun [cited 2022 Jun 23];59(2). Available from: https://pubmed.ncbi.nlm.nih.gov/20179415/10.2332/allergolint.09-RAI-0162 20179415

[76] Hammad H, Lambrecht BN. Dendritic cells and epithelial cells: linking innate and adaptive immunity in asthma. Nat Rev Immunol. 2008;8(3):193–204. 18301423 10.1038/nri2275

[77] Athari SS, Athari SM, Beyzay F, Movassaghi M, Mortaz E, Taghavi M. Critical role of Toll-like receptors in pathophysiology of allergic asthma. Eur J Pharmacol. 2017;808:21–7. 27894811 10.1016/j.ejphar.2016.11.047

[78] Arenas-Padilla M, Duarte‐Gutiérrez JL, Mata‐Haro V. *Bifidobacterium animalis* ssp. *Lactis* Bb12 induces IL‐10 through cell membrane‐associated components via TLR2 in swine. J Appl Microbiol. 2018;125(6):1881–9. 30106205 10.1111/jam.14069 PMC7166459

[79] Lee J, Jung I, Choi JW, Lee CW, Cho S, Choi TG, et al. Micronized and heat-treated *Lactobacillus plantarum* LM1004 stimulates host immune responses via the TLR-2/MAPK/NF-κB signalling pathway *in vitro* and *in vivo*. J Microbiol Biotechnol. 2019;29(5):704–12. 30982316 10.4014/jmb.1812.12059

[80] Vizoso Pinto MG, Rodriguez Gómez M, Seifert S, Watzl B, Holzapfel WH, Franz CMAP. Lactobacilli stimulate the innate immune response and modulate the TLR expression of HT29 intestinal epithelial cells *in vitro*. Int J Food Microbiol. 2009;133(1–2):86–93. 19523707 10.1016/j.ijfoodmicro.2009.05.013

[81] Cui XY, Chen X, Yu CJ, Yang J, Lin ZP, Yin M, et al. Increased expression of Toll-Like receptors 2 and 4 and related cytokines in persistent allergic rhinitis. Otolaryngology–Head Neck Surg. 2015;152(2):233–8. 10.1177/0194599814562173 25505260

[82] Fransson M, Adner M, Erjefält J, Jansson L, Uddman R, Cardell LO. Up-regulation of Toll-like receptors 2, 3 and 4 in allergic rhinitis. Respir Res. 2005;6(1):100. 16146574 10.1186/1465-9921-6-100 PMC1243240

[83] Wenger M, Grosse-Kathoefer S, Kraiem A, Pelamatti E, Nunes N, Pointner L, et al. When the allergy alarm bells toll: the role of Toll-like receptors in allergic diseases and treatment. Front Mol Biosci. 2023. 10.3389/fmolb.2023.1204025. 37426425 10.3389/fmolb.2023.1204025 PMC10325731

[84] Zhong. Upregulation of the expression of Toll-like receptor 9 in basophils in patients with allergic rhinitis: An enhanced expression by allergens - Zhong – 2021 - Scandinavian Journal of Immunology - Wiley Online Library [Internet]. [cited 2023 Jul 11]. Available from: https://onlinelibrary.wiley.com/doi/10.1111/sji.13003 10.1111/sji.13003 33247440

[85] Wang L, Zhan M, Wang J, Chen D, Zhao N, Wang L, et al. Upregulated expression of toll-like receptor 7 in peripheral blood basophils of patients with allergic rhinitis. Am J Rhinol Allergy. 2021;35(6):746–60. 33557582 10.1177/1945892421993034

[86] Gou HZ, Zhang YL, Ren LF, Li ZJ, Zhang L. How do intestinal probiotics restore the intestinal barrier? Front Microbiol. 2022;13:929346. 35910620 10.3389/fmicb.2022.929346 PMC9330398

[87] Zhang Z, Zhang H, Chen T, Shi L, Wang D, Tang D. Regulatory role of short-chain fatty acids in inflammatory bowel disease. Cell Commun Signal. 2022;20(1):64. 35546404 10.1186/s12964-022-00869-5 PMC9097439

[88] Cañas MA, Fábrega MJ, Giménez R, Badia J, Baldomà L. Outer membrane vesicles from probiotic and commensal *Escherichia coli* activate NOD1-mediated immune responses in intestinal epithelial cells. Front Microbiol. 2018. 10.3389/fmicb.2018.00498. 29616010 10.3389/fmicb.2018.00498 PMC5869251

[89] Chin AI, Dempsey PW, Bruhn K, Miller JF, Xu Y, Cheng G. Involvement of receptor-interacting protein 2 in innate and adaptive immune responses. Nature. 2002;416(6877):190–4. 11894097 10.1038/416190a

[90] Raftar SKA, Ashrafian F, Abdollahiyan S, Yadegar A, Moradi HR, Masoumi M, et al. The anti-inflammatory effects of *Akkermansia muciniphila* and its derivates in HFD/CCL4-induced murine model of liver injury. Sci Rep. 2022;12(1):2453. 35165344 10.1038/s41598-022-06414-1 PMC8844054

[91] Kurata A, Yamasaki-Yashiki S, Imai T, Miyazaki A, Watanabe K, Uegaki K. Enhancement of IgA production by membrane vesicles derived from *bifidobacterium longum* subsp. Infantis. Biosci Biotechnol Biochem. 2022;87(1):119–28. 36331264 10.1093/bbb/zbac172

[92] Rabiei N, Ahmadi Badi S, Ettehad Marvasti F, Nejad Sattari T, Vaziri F, Siadat SD. Induction effects of *Faecalibacterium prausnitzii* and its extracellular vesicles on toll-like receptor signaling pathway gene expression and cytokine level in human intestinal epithelial cells. Cytokine. 2019;121:154718. 31153056 10.1016/j.cyto.2019.05.005

[93] Stanton BA. Extracellular vesicles and host–pathogen interactions: a review of inter-kingdom signaling by small noncoding RNA. Genes. 2021;12(7):1010. 34208860 10.3390/genes12071010 PMC8303656

[94] Fukuoka A, Yoshimoto T. Barrier dysfunction in the nasal allergy. Allergol Int. 2018;67(1):18–23. 29150353 10.1016/j.alit.2017.10.006

[95] Gon Y, Hashimoto S. Role of airway epithelial barrier dysfunction in pathogenesis of asthma. Allergol Int. 2018;67(1):12–7. 28941636 10.1016/j.alit.2017.08.011

[96] Capaldo CT, Nusrat A. Cytokine regulation of tight junctions. Biochimica et biophysica acta (BBA). - Biomembr. 2009;1788(4):864–71. 10.1016/j.bbamem.2008.08.027 PMC2699410 18952050

[97] Liu Q, Yu Z, Tian F, Zhao J, Zhang H, Zhai Q, et al. Surface components and metabolites of probiotics for regulation of intestinal epithelial barrier. Microb Cell Fact. 2020;19(1):23. 32024520 10.1186/s12934-020-1289-4 PMC7003451

[98] Fábrega MJ, Aguilera L, Giménez R, Varela E, Alexandra Cañas M, Antolín M et al. Activation of Immune and Defense Responses in the Intestinal Mucosa by Outer Membrane Vesicles of Commensal and Probiotic Escherichia coli Strains. Frontiers in Microbiology [Internet]. 2016 [cited 2022 Apr 13];7. Available from: https://www.frontiersin.org/article/10.3389/fmicb.2016.00705 10.3389/fmicb.2016.00705 PMC4863414 27242727

[99] Tang B, Tang F, Wang Z, Qi G, Liang X, Li B, et al. Upregulation of Akt/NF-κB-regulated inflammation and Akt/Bad-related apoptosis signaling pathway involved in hepatic carcinoma process: suppression by carnosic acid nanoparticle. Int J Nanomed. 2016;11:6401–20. 10.2147/IJN.S101285 PMC5138024 27942213

[100] Lu N, Malemud CJ. Extracellular signal-regulated kinase: a regulator of cell growth, inflammation, chondrocyte and bone cell receptor-mediated gene expression. Int J Mol Sci. 2019;20(15):3792. 31382554 10.3390/ijms20153792 PMC6696446

[101] Jafari et al. Evaluation of the effects of extracellular vesicles derived from Faecalibacterium prausnitzii on lung cancer cell line | SpringerLink [Internet]. 2019 [cited 2022 Jun 21]. Available from: https://link.springer.com/article/10.2478/s11756-019-00229-8

[102] Bourdin A, Gras D, Vachier I, Chanez P. Upper airway x 1: allergic rhinitis and asthma: united disease through epithelial cells. Thorax. 2009;64(11):999–1004. 19864543 10.1136/thx.2008.112862

[103] Saradna A, Do DC, Kumar S, Fu QL, Gao P. Macrophage polarization and allergic asthma. Transl Res. 2018;191:1–14. 29066321 10.1016/j.trsl.2017.09.002 PMC5776696

[104] Wilson RH, Maruoka S, Whitehead GS, Foley JF, Flake GP, Sever ML, et al. The toll-like receptor 5 ligand Flagellin promotes asthma by priming allergic responses to indoor allergens. Nat Med. 2012;18(11):1705–10. 23064463 10.1038/nm.2920 PMC3493750

[105] Trompette A, Divanovic S, Visintin A, Blanchard C, Hegde RS, Madan R, et al. Allergenicity resulting from functional mimicry of a Toll-like receptor complex protein. Nature. 2009;457(7229):585–8. 19060881 10.1038/nature07548 PMC2843411

[106] Raghavan B, Martin SF, Esser PR, Goebeler M, Schmidt M. Metal allergens nickel and cobalt facilitate TLR4 homodimerization independently of MD2. EMBO Rep. 2012;13(12):1109–15. 23059983 10.1038/embor.2012.155 PMC3512400

[107] Royer J, Emara M, Yang C, Al-Ghouleh A, Tighe P, Jones N, et al. The mannose receptor mediates the uptake of diverse native allergens by dendritic cells and determines allergen-induced T cell polarization through modulation of IDO activity. J Immunol. 2010;185(3):1522–31. 20610655 10.4049/jimmunol.1000774

[108] Mir B, Goettsch C. Extracellular vesicles as delivery vehicles of specific cellular cargo. Cells. 2020;9(7):1601. 32630649 10.3390/cells9071601 PMC7407641

[109] Anand D, Chaudhuri A. Bacterial outer membrane vesicles: new insights and applications. Mol Membr Biol. 2016;33(6–8):125–37. 29189113 10.1080/09687688.2017.1400602

[110] Diaz-Garrido N, Badia J, Baldomà L. Modulation of dendritic cells by microbiota extracellular vesicles influences the cytokine profile and exosome cargo. Nutrients. 2022;14(2):344. 35057528 10.3390/nu14020344 PMC8778470

[111] Diaz-Garrido N, Fábrega MJ, Vera R, Giménez R, Badia J, Baldomà L. Membrane vesicles from the probiotic Nissle 1917 and gut resident *Escherichia coli* strains distinctly modulate human dendritic cells and subsequent T cell responses. J Funct Foods. 2019;61:103495.

[112] Hu et al. Probiotic Escherichia coli Nissle 1917-derived outer membrane vesicles enhance immunomodulation and antimicrobial activity in RAW264.7 macrophages | BMC Microbiology | Full Text [Internet]. 2020 [cited 2022 Jun 20]. Available from: https://bmcmicrobiol.biomedcentral.com/articles/10.1186/s12866-020-01953-x 10.1186/s12866-020-01953-x PMC7457259 32854612

[113] Braga M, Quecchia C, Cavallucci E, Di Giampaolo L, Schiavone C, Petrarca C, et al. T regulatory cells in allergy. Int J Immunopathol Pharmacol. 2011;24(1 Suppl):S55–64. 21329567

[114] Elkord E. Role of regulatory T cells in allergy: implications for therapeutic strategy. IADT. 2006;5(4):211–7. 10.2174/187152806779010945 17168791

[115] Calzada D, Baos S, Cremades-Jimeno L, Cárdaba B. Immunological mechanisms in allergic diseases and allergen tolerance: the role of Treg cells. J Immunol Res. 2018;2018:1–10. 10.1155/2018/6012053 PMC6022267 30013991

[116] González-de-Olano D, Álvarez-Twose I. Mast cells as key players in allergy and inflammation. J Investig Allergol Clin Immunol. 2018;28(6):365–78. 30530385 10.18176/jiaci.0327

[117] Zwicker C, Sarate P, Drinić M, Ambroz K, Korb E, Smole U, et al. Prophylactic and therapeutic Inhibition of allergic airway inflammation by probiotic Escherichia coli O83. J Allergy Clin Immunol. 2018;142(6):1987–e19907. 30125662 10.1016/j.jaci.2018.07.029

[118] Razim A, Zabłocka A, Schmid A, Thaler M, Černý V, Weinmayer T, et al. Bacterial extracellular vesicles as intranasal postbiotics: detailed characterization and interaction with airway cells. J Extracell Vesicles. 2024;13(10):e70004. 39429019 10.1002/jev2.70004 PMC11491762

[119] Sim S, Park HJ, Kim YK, Choi Y, Park HS. *Lactobacillus paracasei*-derived extracellular vesicles alleviate neutrophilic asthma by inhibiting the JNK pathway in airway epithelium. Allergol Int. 2024;73(2):302–12. 37953104 10.1016/j.alit.2023.10.008

[120] Lee DH, Park HK, Lee HR, Sohn H, Sim S, Park HJ, et al. Immunoregulatory effects of *Lactococcus lactis*-derived extracellular vesicles in allergic asthma. Clin Transl Allergy. 2022;12(3):e12138. 35344296 10.1002/clt2.12138 PMC8967260

[121] Martínez-Ruiz S, Sáez-Fuertes L, Casanova-Crespo S, Rodríguez-Lagunas MJ, Pérez-Cano FJ, Badia J, et al. Microbiota-derived extracellular vesicles promote immunity and intestinal maturation in suckling rats. Nutrients. 2023;15(21):4701. 37960354 10.3390/nu15214701 PMC10649425

[122] Pantazi AC, Mihai CM, Balasa AL, Chisnoiu T, Lupu A, Frecus CE, et al. Relationship between gut microbiota and allergies in children: a literature review. Nutrients. 2023;15(11):2529. 37299492 10.3390/nu15112529 PMC10255222

[123] Augustine T, Kumar M, Al Khodor S, van Panhuys N. Microbial dysbiosis tunes the immune response towards allergic disease outcomes. Clin Rev Allerg Immunol. 2023;65(1):43–71. 10.1007/s12016-022-08939-9 PMC10326151 35648372

[124] Ouwehand AC, Nermes M, Collado MC, Rautonen N, Salminen S, Isolauri E. Specific probiotics alleviate allergic rhinitis during the Birch pollen season. WJG. 2009;15(26):3261. 19598302 10.3748/wjg.15.3261 PMC2710782

[125] Feng S, Liu Y, Xu J, Fan J, Li J, Wu Z, et al. Three strains of *Lactobacillus* derived from piglets alleviated intestinal oxidative stress induced by Diquat through extracellular vesicles. Nutrients. 2023;15(19):4198. 37836484 10.3390/nu15194198 PMC10574712

[126] Xu J, Ye Y, Ji J, Sun J, Wang JS, Sun X. Untargeted metabolomic profiling reveals changes in gut microbiota and mechanisms of its regulation of allergy in OVA-sensitive BALB/c mice. J Agric Food Chem. 2022;70(10):3344–56. 35232013 10.1021/acs.jafc.1c07482

[127] Zhang J, Ma JY, Li QH, Su H, Sun X. *Lactobacillus rhamnosus* GG induced protective effect on allergic airway inflammation is associated with gut microbiota. Cell Immunol. 2018;332:77–84. 30097177 10.1016/j.cellimm.2018.08.002

[128] Yoon et al. Akkermansia muciniphila secretes a glucagon-like peptide-1-inducing protein that improves glucose homeostasis and ameliorates metabolic disease in mice | Nature Microbiology [Internet]. 2021 [cited 2022 May 2]. Available from: https://www.nature.com/articles/s41564-021-00880-5 10.1038/s41564-021-00880-5 33820962

[129] Sapugahawatte DN, Godakumara K, Mäesaar M, Ekanayake G, Midekessa GB, Prasadani M, et al. Harnessing nature’s defence: the antimicrobial efficacy of pasteurised cattle Milk-Derived extracellular vesicles on Staphylococcus aureus ATCC 25923. Int J Mol Sci. 2024;25(9):4759. 38731976 10.3390/ijms25094759 PMC11083917

[130] Proksch E, Brandner JM, Jensen JM. The skin: an indispensable barrier. Exp Dermatol. 2008;17(12):1063–72. 19043850 10.1111/j.1600-0625.2008.00786.x

[131] Elias PM, Wakefield JS. Mechanisms of abnormal lamellar body secretion and the dysfunctional skin barrier in patients with atopic dermatitis. J Allergy Clin Immunol. 2014;134(4):781–e7911. 25131691 10.1016/j.jaci.2014.05.048 PMC4186911

[132] Palomares O, Akdis M, Martín-Fontecha M, Akdis CA. Mechanisms of immune regulation in allergic diseases: the role of regulatory T and B cells. Immunol Rev. 2017;278(1):219–36. 28658547 10.1111/imr.12555

[133] Akdis CA, Akdis M. Mechanisms of allergen-specific immunotherapy. J Allergy Clin Immunol. 2011;127(1):18–27. quiz 28–9. 21211639 10.1016/j.jaci.2010.11.030

[134] Galli SJ, Tsai M. IgE and mast cells in allergic disease. Nat Med. 2012;18(5):693–704. 22561833 10.1038/nm.2755 PMC3597223

[135] Galli SJ, Tsai M, Piliponsky AM. The development of allergic inflammation. Nature. 2008;454(7203):445–54. 18650915 10.1038/nature07204 PMC3573758

[136] Pascal M, Perez-Gordo M, Caballero T, Escribese MM, Lopez Longo MN, Luengo O et al. Microbiome and Allergic Diseases. Front Immunol [Internet]. 2018 Jul 17 [cited 2025 Feb 17];9. Available from: https://www.frontiersin.org/journals/immunology/articles/10.3389/fimmu.2018.01584/full 10.3389/fimmu.2018.01584 PMC6056614 30065721

[137] Pandiyan P, Bhaskaran N, Zou M, Schneider E, Jayaraman S, Huehn J. Microbiome Dependent Regulation of Tregs and Th17 Cells in Mucosa. Front Immunol [Internet]. 2019 Mar 8 [cited 2025 Feb 17];10. Available from: https://www.frontiersin.org/journals/immunology/articles/10.3389/fimmu.2019.00426/full 10.3389/fimmu.2019.00426 PMC6419713 30906299

[138] Sharma G, Im SH. Probiotics as a potential immunomodulating pharmabiotics in allergic diseases: current status and future prospects. Allergy Asthma Immunol Res. 2018;10(6):575–90. 30306743 10.4168/aair.2018.10.6.575 PMC6182196

[139] Yang T, Martin P, Fogarty B, Brown A, Schurman K, Phipps R, et al. Exosome delivered anticancer drugs across the blood-brain barrier for brain cancer therapy in *Danio rerio*. Pharm Res. 2015;32(6):2003–14. 25609010 10.1007/s11095-014-1593-y PMC4520542

[140] Watkins HC, Rappazzo CG, Higgins JS, Sun X, Brock N, Chau A, et al. Safe recombinant outer membrane vesicles that display M2e elicit heterologous influenza protection. Mol Ther. 2017;25(4):989–1002. 28215994 10.1016/j.ymthe.2017.01.010 PMC5383554

[141] Richter M, Vader P, Fuhrmann G. Approaches to surface engineering of extracellular vesicles. Adv Drug Deliv Rev. 2021;173:416–26. 33831479 10.1016/j.addr.2021.03.020

[142] Haney MJ, Klyachko NL, Zhao Y, Gupta R, Plotnikova EG, He Z, et al. Exosomes as drug delivery vehicles for Parkinson’s disease therapy. J Controlled Release. 2015;207:18–30. 10.1016/j.jconrel.2015.03.033 PMC4430381 25836593

[143] Kooijmans SAA, Fliervoet LAL, van der Meel R, Fens MHAM, Heijnen HFG, van Bergen PMP, et al. PEGylated and targeted extracellular vesicles display enhanced cell specificity and circulation time. J Controlled Release. 2016;224:77–85. 10.1016/j.jconrel.2016.01.009 26773767

[144] Welsh JA, Goberdhan DCI, O’Driscoll L, Buzas EI, Blenkiron C, Bussolati B, et al. Minimal information for studies of extracellular vesicles (MISEV2023): from basic to advanced approaches. J Extracell Vesicles. 2024;13(2):e12404. 38326288 10.1002/jev2.12404 PMC10850029

[145] Van Deun J, Mestdagh P, Agostinis P, Akay Ö, Anand S, Anckaert J, et al. EV-TRACK: transparent reporting and centralizing knowledge in extracellular vesicle research. Nat Methods. 2017;14(3):228–32. 28245209 10.1038/nmeth.4185

[146] Holcar M, Kandušer M, Lenassi M. Blood Nanoparticles – Influence on Extracellular Vesicle Isolation and Characterization. Front Pharmacol [Internet]. 2021 Nov 10 [cited 2024 Sep 2];12. Available from: https://www.frontiersin.org/journals/pharmacology/articles/10.3389/fphar.2021.773844/full 10.3389/fphar.2021.773844 PMC8635996 34867406

[147] Ou Z, Situ B, Huang X, Xue Y, He X, Li Q, et al. Single-particle analysis of circulating bacterial extracellular vesicles reveals their biogenesis, changes in blood and links to intestinal barrier. J Extracell Vesicles. 2023;12(12):e12395. 38050834 10.1002/jev2.12395 PMC10696524

